# Postoperative risk of IDH-mutant glioma–associated seizures and their potential management with IDH-mutant inhibitors

**DOI:** 10.1172/JCI168035

**Published:** 2023-06-15

**Authors:** Michael R. Drumm, Wenxia Wang, Thomas K. Sears, Kirsten Bell-Burdett, Rodrigo Javier, Kristen Y. Cotton, Brynna Webb, Kayla Byrne, Dusten Unruh, Vineeth Thirunavu, Jordain Walshon, Alicia Steffens, Kathleen McCortney, Rimas V. Lukas, Joanna J. Phillips, Esraa Mohamed, John D. Finan, Lucas Santana-Santos, Amy B. Heimberger, Colin K. Franz, Jonathan Kurz, Jessica W. Templer, Geoffrey T. Swanson, Craig Horbinski

**Affiliations:** 1 Department of Neurological Surgery and; 2 Department of Preventive Medicine, Northwestern University, Chicago, Illinois, USA.; 3 University of Chicago Pritzker School of Medicine, Chicago, Illinois, USA.; 4 University of Illinois at Chicago, Chicago, Illinois, USA.; 5 Department of Pharmacology, Northwestern University, Chicago, Illinois, USA.; 6 Northwestern University, Evanston, Illinois, USA.; 7 Bio-Techne, Minneapolis, Minnesota, USA.; 8 Ken & Ruth Davee Department of Neurology and; 9 Lou and Jean Malnati Brain Tumor Institute of the Robert H. Lurie Comprehensive Cancer Center, Northwestern University, Chicago, Illinois, USA.; 10 Department of Neurological Surgery, Brain Tumor Center, UCSF, San Francisco, California, USA.; 11 Department of Mechanical and Industrial Engineering, University of Illinois at Chicago, Chicago, Illinois, USA.; 12 Department of Pathology and; 13 Department of Physical Medicine and Rehabilitation, Northwestern University, Chicago, Illinois, USA.; 14 Biologics Laboratory, Shirley Ryan AbilityLab, Chicago, Illinois, USA.; 15 Merck & Co. Inc., North Wales, Pennsylvania, USA.

**Keywords:** Neuroscience, Oncology, Brain cancer, Epilepsy, Seizures

## Abstract

Seizures are a frequent complication of adult-type diffuse gliomas, and are often difficult to control with medications. Gliomas with mutations in isocitrate dehydrogenase 1 or 2 (IDHmut) are more likely than IDH–wild type (IDHwt) gliomas to cause seizures as part of their initial clinical presentation. However, whether IDHmut is also associated with seizures during the remaining disease course, and whether IDHmut inhibitors can reduce seizure risk, are unclear. Clinical multivariable analyses showed that preoperative seizures, glioma location, extent of resection, and glioma molecular subtype (including IDHmut status) all contributed to postoperative seizure risk in adult-type diffuse glioma patients, and that postoperative seizures were often associated with tumor recurrence. Experimentally, the metabolic product of IDHmut, d-2-hydroxyglutarate, rapidly synchronized neuronal spike firing in a seizure-like manner, but only when non-neoplastic glial cells were present. In vitro and in vivo models recapitulated IDHmut glioma–associated seizures, and IDHmut inhibitors currently being evaluated in glioma clinical trials inhibited seizures in those models, independent of their effects on glioma growth. These data show that postoperative seizure risk in adult-type diffuse gliomas varies in large part by molecular subtype, and that IDHmut inhibitors could play a key role in mitigating such risk in IDHmut glioma patients.

## Graphical abstract

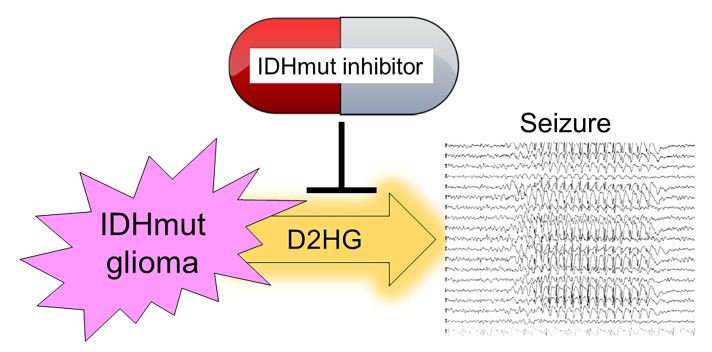

## Introduction

Most patients with adult-type diffusely infiltrative gliomas will experience at least one seizure during their disease course (1, 2). Seizures are often part of the clinical presentation for these tumors, and can persist after surgery and adjuvant therapy. This condition, called tumor-associated epilepsy (TAE), not only impairs quality of life, but can even manifest as life-threatening status epilepticus (3). Seizures cause hippocampal sclerosis, excitotoxic loss of neurons, inflammation, and oxidative stress (4–7). Thus, patients with TAE suffer from reduced cognition, memory, and executive functioning (8). While there are numerous antiseizure medications (ASMs) that treat most forms of epilepsy, 30%–60% of TAE patients experience breakthrough seizures while on levetiracetam (Keppra), a common first-line ASM, and require second-line ASMs that have adverse side effects (9–13). Moreover, increased neuronal activity is now known to stimulate glioma proliferation and infiltration (14–19). A better understanding of how gliomas induce seizures, and how to control them more effectively, is therefore needed.

Mutations in *isocitrate dehydrogenase 1* (*IDH1*) or *IDH2* (“IDHmut”) occur in approximately 30% of adult-type diffusely infiltrative gliomas (20). We and others previously showed that most patients with IDHmut gliomas have seizures as part of their initial presentation, even more than patients with IDH–wild type (IDHwt) glioblastomas (1, 21–23). Whereas wild-type IDH1 and IDH2 enzymes oxidize isocitrate into α-ketoglutarate, mutant IDH1 and IDH2 reduce α-ketoglutarate to d-2-hydroxyglutarate (D2HG). As a result, D2HG concentration is elevated 10- to 100-fold in IDHmut gliomas compared with IDHwt gliomas (24). D2HG is exported out of IDHmut glioma cells, and likely reaches low millimolar concentrations within the tumor microenvironment (25).

We previously showed that D2HG increases the excitability of cultured neurons (1), providing a direct mechanistic link between IDHmut glioma and TAE. In the current study, we extended those findings into new in vitro and in vivo models, including determining the potential of IDHmut enzyme inhibitors, which are currently being evaluated in IDHmut glioma patients (26, 27), to control TAE. We also studied patterns of seizure recurrence during the entire course of disease in adult-type diffuse glioma patients, determining which variables, including glioma genotype, contribute to postoperative TAE risk.

## Results

### Variables affecting postoperative seizure risk include IDHmut.

To study patterns of postoperative glioma-associated seizures, we performed a retrospective observational survey of 247 consecutive patients with newly diagnosed World Health Organization (WHO) grade 2–4 adult-type diffuse gliomas. Median follow-up was 20.3 months for all patients (IQR 10.7–35.0 months) (Table 1). Forty-seven patients had IDHmut astrocytoma, 32 patients had IDHmut and 1p/19q–codeleted oligodendroglioma, and 168 patients had IDHwt glioblastoma, with median (IQR) follow-up of 31.8 (23.5–46.4) months, 37.6 (29.9–45.2) months, and 15.0 (7.8–24.0) months, respectively. Consistent with our prior study (1), 60 of 79 (76%) patients with an IDHmut glioma experienced at least 1 seizure as part of their initial preoperative clinical presentation, whereas only 75 of 168 (45%) IDHwt glioblastoma patients did (*P* < 0.0001). Among IDHmut gliomas, 33 of 47 (70%) patients with astrocytoma and 27 of 32 (84%) patients with oligodendroglioma had a preoperative seizure (*P* = 0.19).

**Figure 1:**
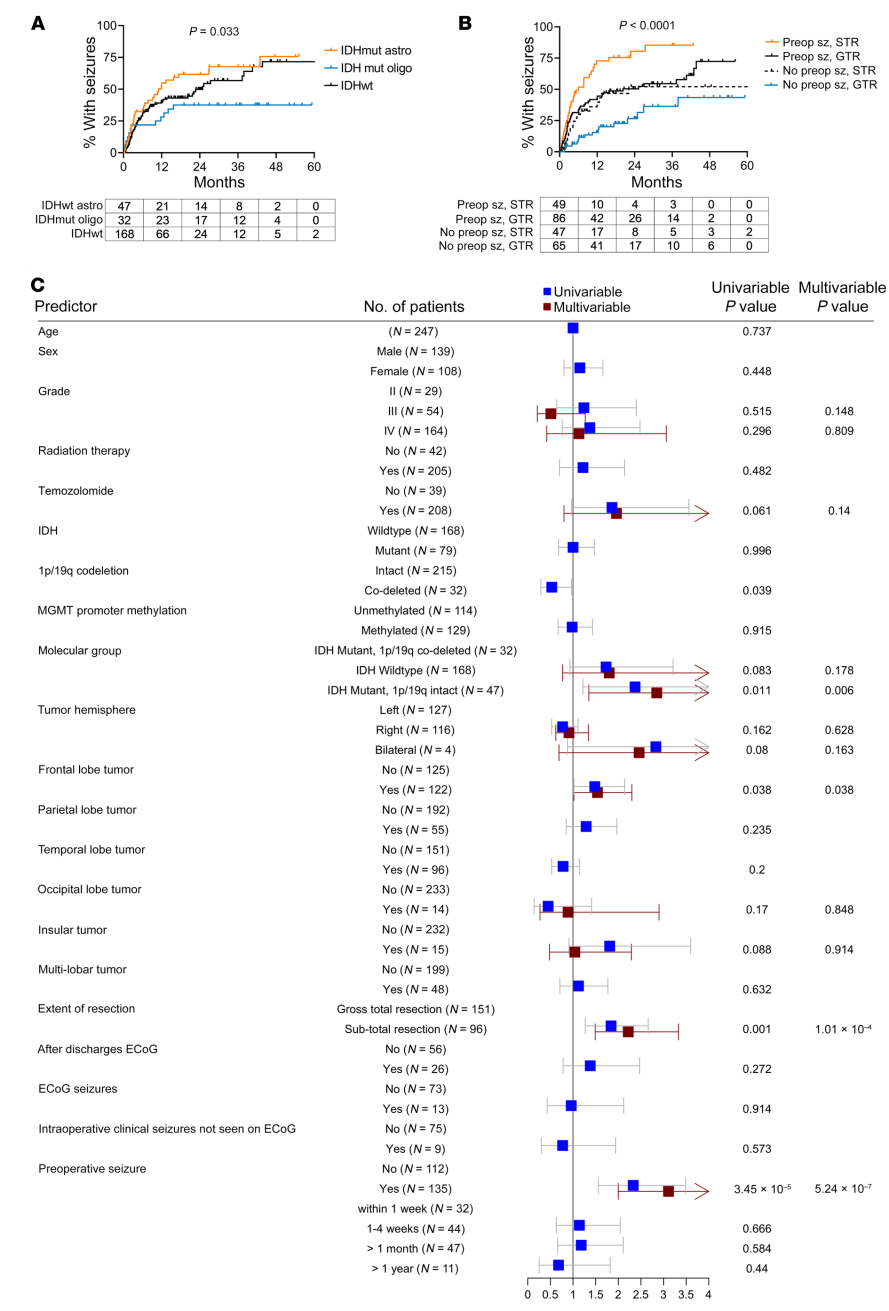
Risk factors for postoperative seizures in patients with diffusely infiltrative glioma. (**A** and **B**) Time-to-event analyses performed for postoperative seizures in patients with adult-type diffusely infiltrative glioma (*n* = 247), stratified by glioma molecular status (IDHmut astrocytoma, *n* = 47; IDHwt glioblastoma, *n* = 168; IDHmut oligodendroglioma, *n* = 32) (**A**) or by whether patients experienced preoperative seizures (Preop sz) and/or subtotal resection (STR) (preoperative seizure and STR, *n* = 49; preoperative seizure and gross total resection [GTR], *n* = 86; no preoperative seizure and STR, *n* = 47; no preoperative seizure and GTR, *n* = 65) (**B**). Data were analyzed with the log-rank (Mantel-Cox) test. (**C**) Forest plot depicting univariate and multivariate analyses of risk factors associated with postoperative seizures. ECoG, electrocorticography.

In postoperative time-to-seizure analyses, patients with IDHmut astrocytomas had a median time to first postoperative seizure of 11.0 months, followed by IDHwt glioblastoma patients (24.9 months) and IDHmut, 1p/19q–codeleted oligodendroglioma patients (median not reached) (*P* = 0.033; Figure 1A). Those who had a preoperative seizure developed postoperative seizures faster than patients who did not have a preoperative seizure (median = 10.7 months vs. not reached, *P* < 0.0001; Supplemental Figure 1A; supplemental material available online with this article; https://doi.org/10.1172/JCI168035DS1), regardless of whether the glioma was IDHmut or IDHwt (Supplemental Figure 1, B and C). Likewise, those with subtotal resection (STR) of their glioma showed faster time to seizure than those with gross total resection (GTR) (median = 10.1 months vs. 37.9 months, *P* = 0.001; Supplemental Figure 1D), although this was statistically significant only for IDHmut gliomas (median = 9.9 months vs. not reached, *P* = 0.005; Supplemental Figure 1E), not IDHwt glioblastomas (median = 9.5 months vs. 25.3 months, *P* = 0.20; Supplemental Figure 1F). Overall, patients with preoperative seizures and STR had the fastest time to first postoperative seizure (*n* = 49, median = 6.0 months), while patients who had no preoperative seizures and had GTR were the slowest to develop postoperative seizures (*n* = 65, median not reached) (*P* < 0.0001; Figure 1B), regardless of IDHmut status (Supplemental Figure 1, G and H).

Univariable and multivariable analyses using Cox proportional hazards modeling showed that IDHmut, 1p/19q–intact astrocytomas (hazard ratio [HR] 2.85, 95% confidence interval [CI] 1.35–6.01, *P* = 0.006), frontal lobe involvement of tumor (HR 1.54, 95% CI 1.02–2.30, *P* = 0.038), STR (HR 2.22, 95% CI 1.49–3.33, *P* < 0.0001), and preoperative seizures (HR 3.13, 95% CI 2.00–4.84, *P* < 0.0001) were all significant independent correlates with postoperative seizure risk (Figure 1C and Table 2).

### The timing and nature of postoperative seizures are a harbinger of glioma recurrence and progression.

**Figure 2:**
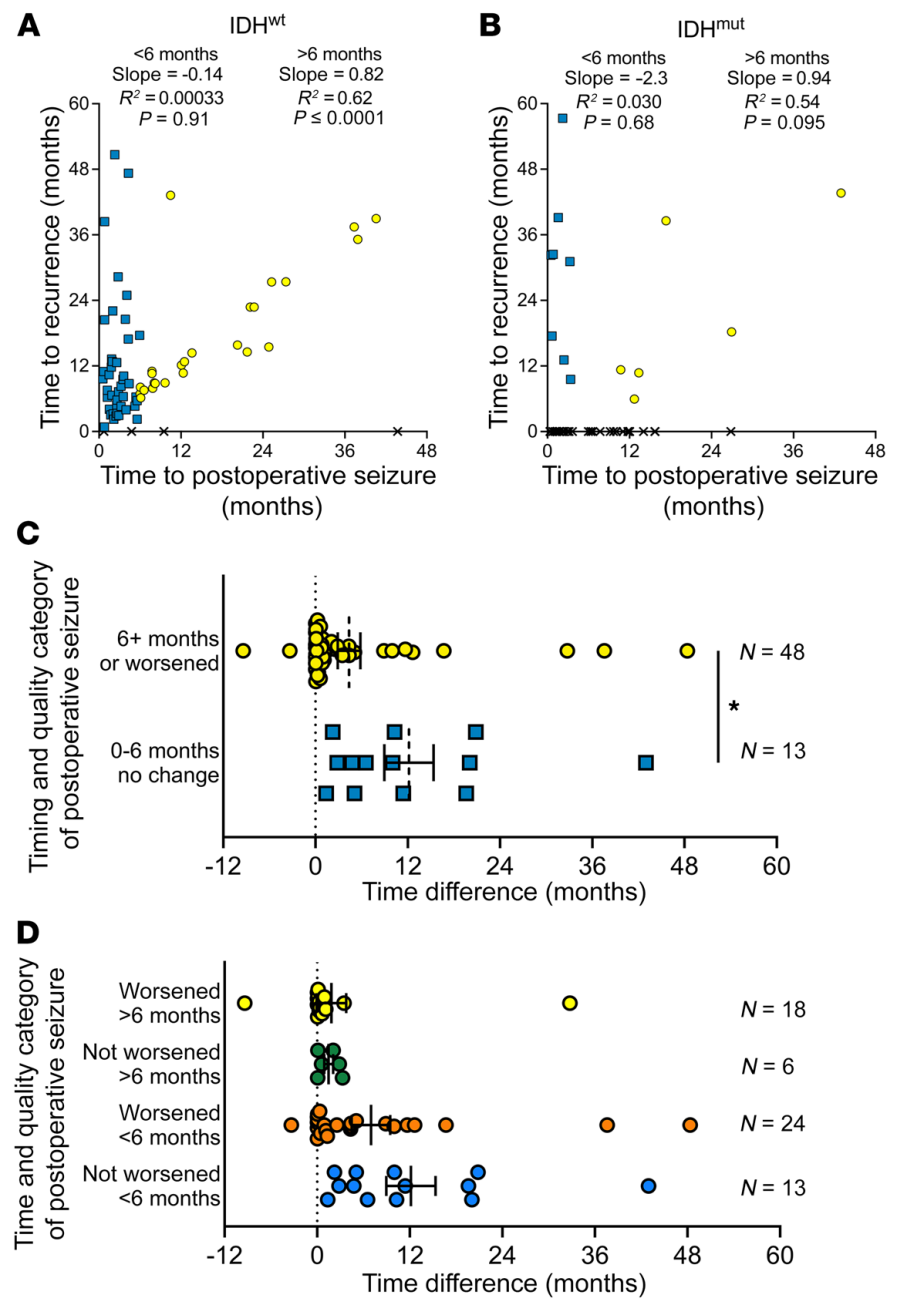
Postoperative seizures relative to recurrence in adult-type diffuse glioma patients. (**A** and **B**) Time to first postoperative seizure and time to recurrence were graphed for patients with postoperative seizures with IDHwt glioblastoma (*n* = 72) (**A**) and IDHmut glioma (*n* = 43) (**B**). If a patient did not experience recurrence during their clinical follow-up, an X was plotted on the *x* axis at the time of their postoperative seizure. Patients with postoperative seizures before 6 months are indicated with blue squares; patients with postoperative seizures on or after 6 months are indicated with yellow circles. Data were analyzed with linear regression. (**C** and **D**) For patients with IDHwt glioblastoma who had a postoperative seizure that was not caused by second-line treatment (*n* = 61), the difference in time between a patient’s postoperative seizure and tumor recurrence was calculated. Positive values indicate that the postoperative seizure preceded tumor recurrence. Bars represent mean ± SEM. *P < 0.05 by unpaired, 2-tailed *t* test.

Among 72 patients with IDHwt glioblastoma who had postoperative seizures, 68 (94%) had experienced a recurrence by last follow-up, while of the 43 patients with IDHmut glioma who had postoperative seizures, only 14 (33%) had a recurrence within the study time frame. Among IDHwt glioblastoma patients, the first postoperative seizure showed a strong correlation with tumor recurrence only if it arose after a 6-month post-surgical window, not before (Figure 2A). A similar pattern was apparent with IDHmut glioma patients, though not enough clinical recurrences had yet occurred to reach statistical significance (Figure 2B). Among 45 IDHwt glioblastoma patients who experienced their first seizure within 6 months of surgery, 15 (33%) also had tumor recurrence within 6 months. In contrast, none of the 23 IDHmut glioma patients who had a seizure within 6 months of surgery had a tumor recurrence within that time frame (*P* = 0.001).

Another way to classify postoperative seizures is whether the postoperative seizure was similar to the preoperative seizure that the patient experienced, or whether it worsened (see Methods and Supplemental Figure 2). To explore this further, IDHwt patients were stratified into 2 groups: (a) those who had their seizure after 6 months following resection and/or their seizure worsened compared with the preoperative seizure (*n* = 48); and (b) those who had a seizure before 6 months that was the same as or milder than the preoperative seizure (*n* = 13). Next, the time to postoperative seizure was subtracted from time to tumor progression to see how closely those 2 events aligned (Figure 2, C and D). Among the 48 patients with a late and/or a worse postoperative seizure, overt clinical tumor progression occurred a median of only 0.80 months later (IQR 0.066–4.12). Of those 48 patients, 46 (96%) had that seizure before tumor progression, while 2 had their seizures shortly after progression had already been clinically established. In contrast, a postoperative seizure that occurred within 6 months of initial surgery, and did not worsen in quality relative to earlier seizures, preceded frank glioma progression by a median of 10.0 months (IQR 3.80–19.84 months). This extended latency was significantly longer than in patients with late and/or worsened postoperative seizures (*P* = 0.021). These data therefore suggest that seizures may portend clinical recurrence of adult-type diffuse gliomas, but only if they manifest at least 6 months after initial surgery, or begin less than 6 months after surgery but are qualitatively worse than they were preoperatively.

### D2HG triggers seizure-like neuronal activity in a glia-dependent manner.

**Figure 3:**
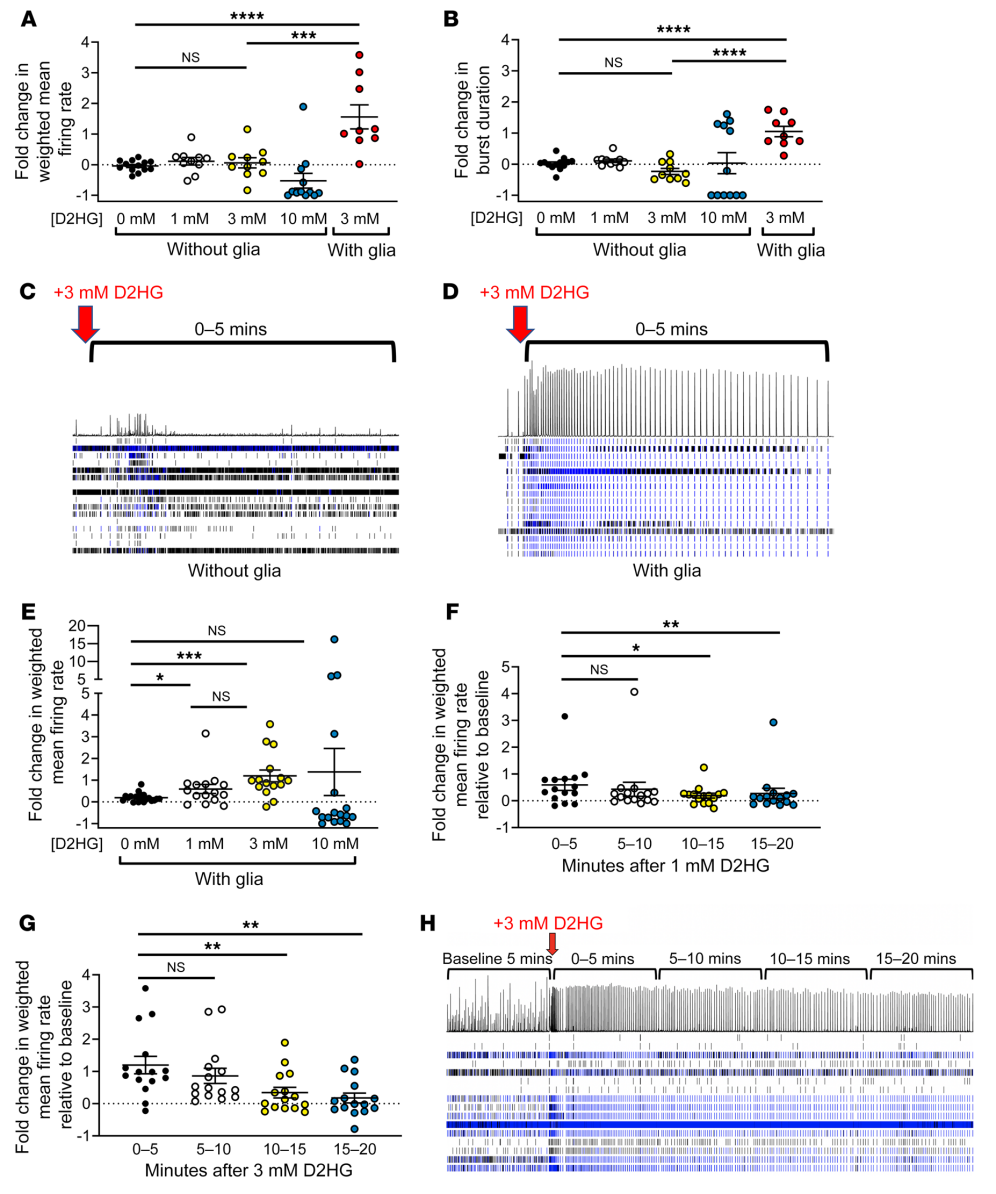
D2HG requires glial cells to trigger neuronal firing in vitro. (**A** and **B**) Day 21 mouse neurons cultured with or without glia were recorded on a multi-electrode plate for 5 minutes, and then treated with vehicle or 1, 3, or 10 mM D2HG (vehicle –glia, *n* = 13; 1 mM D2HG +glia, *n* = 10; 3 mM D2HG –glia, *n* = 10; 10 mM D2HG –glia, *n* = 12; 3 mM D2HG +glia, *n* = 9). Experiment was repeated twice for a total of 3 times. Fold change after treatment was calculated for each well. Bars represent mean ± SEM. ***P = 0.002, ****P < 0.0001 by unpaired, 2-tailed *t* test (Bonferroni-adjusted *P* = 0.006). (**C** and **D**) Raster plots of wells with neurons only (**C**) or neurons with glia (**D**) treated with 3 mM D2HG at the time point indicated by the red arrows. Horizontal rows indicate firing activity of each electrode in a well (16 electrodes per well). Histogram above the rows depicts the summation of the firing activity of all electrodes in the well at a given time point. (**E**) Day 21 mouse neurons cultured with glia had baseline weighted mean firing rate recorded on a multi-electrode well plate for 5 minutes, then were treated with vehicle or 1, 3, or 10 mM D2HG (vehicle, *n* = 19; 1 mM D2HG, *n* = 15; 3 mM D2HG, *n* = 15; 10 mM D2HG, *n* = 17). Fold change of weighted mean firing rate for 5 minutes after treatment was calculated for each well. Bars represent mean ± SEM. *P < 0.05, ***P < 0.001 by 2-tailed *t* test. Experiment was repeated twice for a total of 3 times. (**F** and **G**) Neurons cultured with glia were recorded for 20 minutes following treatment with either 1 mM D2HG (*n* = 15) (**F**) or 3 mM D2HG (*n* = 15) (**G**), and fold change of weighted mean firing rate was calculated for each well in 5-minute intervals. Bars represent mean ± SEM. *P < 0.05, **P < 0.01 by unpaired, 2-tailed *t* test. Experiment was repeated twice for a total of 3 times. (**H**) Representative raster plot of well with neurons cultured with glia treated with 3 mM D2HG and recorded for 20 minutes following treatment.

We sought to further explore the connection between IDHmut gliomas, their D2HG product, and seizures. First, we established 21-day-old mouse cortical neuron cultures, in the presence or absence of non-neoplastic glial cells, on multi-electrode array plates (Supplemental Figure 3). Baseline firing metrics and firing patterns and synaptic density under both conditions are shown in Supplemental Figures 4–6. Neither 1 mM nor 3 mM exogenous D2HG changed the weighted mean firing rate of neuron-only cultures (*P* = 0.22–0.52; Figure 3A). In contrast, 3 mM D2HG increased the firing rate of neurons cocultured with glial cells by 56% (*P* < 0.0001 vs. vehicle control, *P* = 0.0020 vs. 3 mM without glia; Figure 3A). As exogenous D2HG exists as a disodium salt (see Methods), 6 mM sodium was separately tested, but did not alter the firing rate of neurons cocultured with glial cells (Supplemental Figure 7). D2HG also increased burst duration in neuron-glia cocultures (*P* < 0.0001 vs. vehicle control or neuron-only cultures; Figure 3, B–D). In the presence of glia, neurons responded to as little as 1 mM D2HG (*P* = 0.044; Figure 3E). These effects were rapid, occurring within 5 minutes of exogenous D2HG application, with firing rates returning to baseline 10–15 minutes after treatment (Figure 3, F and G). Although bulk firing rates normalized, cocultures retained changes in their bursting patterns for at least 20 minutes after 3 mM D2HG (Figure 3H). In those cocultures, 3 mM D2HG also increased interspike interval coefficient of variation (a metric of firing variability; *P* = 0.023), burst duration (*P* = 0.014), network burst duration (*P* = 0.0002), and number of spikes per burst (*P* = 0.023), but not mean interspike interval per burst (*P* = 0.42) or burst frequency (*P* = 0.26) (Supplemental Figure 8). These data demonstrate that D2HG promotes synchronization of neuronal firing into bursts and lengthens the duration of clustered intra-burst spikes, thus mimicking seizures in vitro.

### Effects of D2HG on ionotropic glutamate receptors, glutamate reuptake transporters, and mTOR signaling.

D2HG is structurally similar to the excitatory neurotransmitter glutamate, with a hydroxyl group instead of an amine group at carbon 2 (1). However, D2HG did not activate ionotropic homomeric GluA4(flip) AMPA receptors, GluK2 kainate receptors, GluN1/2A NMDA receptors, or GluN1/2B NMDA receptors (Supplemental Figure 9, A–D). D2HG also did not elicit electrogenic transport current from the excitatory amino acid reuptake transporter GLT1a (Supplemental Figure 9E), suggesting that D2HG does not promote burst firing of neurons via competitive inhibition of synaptic glutamate reuptake. D2HG also did not enhance the effect of dihydrokainate (DHK), a selective GLT1/EAAT2 reuptake inhibitor, on weighted mean firing rate (Supplemental Figure 10A). Instead, the firing pattern of combined DHK plus D2HG resembled both the high firing rate elicited by DHK and the synchronized bursting triggered by D2HG (Supplemental Figure 10, B–E).

The effect of D2HG on mTOR signaling is controversial, with one group showing that it activates mTOR signaling (28) and another group showing that it inhibits mTOR signaling (29). A third group reported that the pro-seizure effects of IDHmut gliomas may be due to D2HG activation of neuronal mTOR (30). In our studies, 3 mM D2HG decreased, rather than increased, in vitro neuronal mTOR pathway activity, as indicated by nuclear phospho-S6 localization (Supplemental Figure 11, A–D). Among patient-derived tissues from newly diagnosed adult-type diffuse gliomas collected and analyzed at 2 separate institutions, phospho-S6 was not significantly different in neurons admixed within IDHwt glioblastoma, IDHmut astrocytoma, or IDHmut and 1p/19q–codeleted oligodendroglioma (*P* = 0.50; Supplemental Figure 11E). There was also no difference in neuronal phospho-S6 according to the presence or absence of preoperative seizures in those cases (*P* = 0.15; Supplemental Figure 11F). Finally, because synchronized bursting activity occurred rapidly following application of exogenous D2HG to neuron-glia cocultures (Figure 3, D and E), we assessed phospho-S6 expression within the first 5 minutes after treatment of exogenous D2HG. Phospho-S6 did not increase after 5 minutes of treatment with either 1 mM D2HG or 3 mM D2HG in either neuron-glia cocultures or neuron-only cultures (Supplemental Figure 11G). Thus, the excitatory effects of D2HG may not be directly through ionotropic glutamate receptors, glutamate reuptake transporters, or the mTOR pathway.

### IDHmut inhibitors block the excitatory effect of patient-derived IDHmut gliomas in 3D human cortical spheroids.

**Figure 4:**
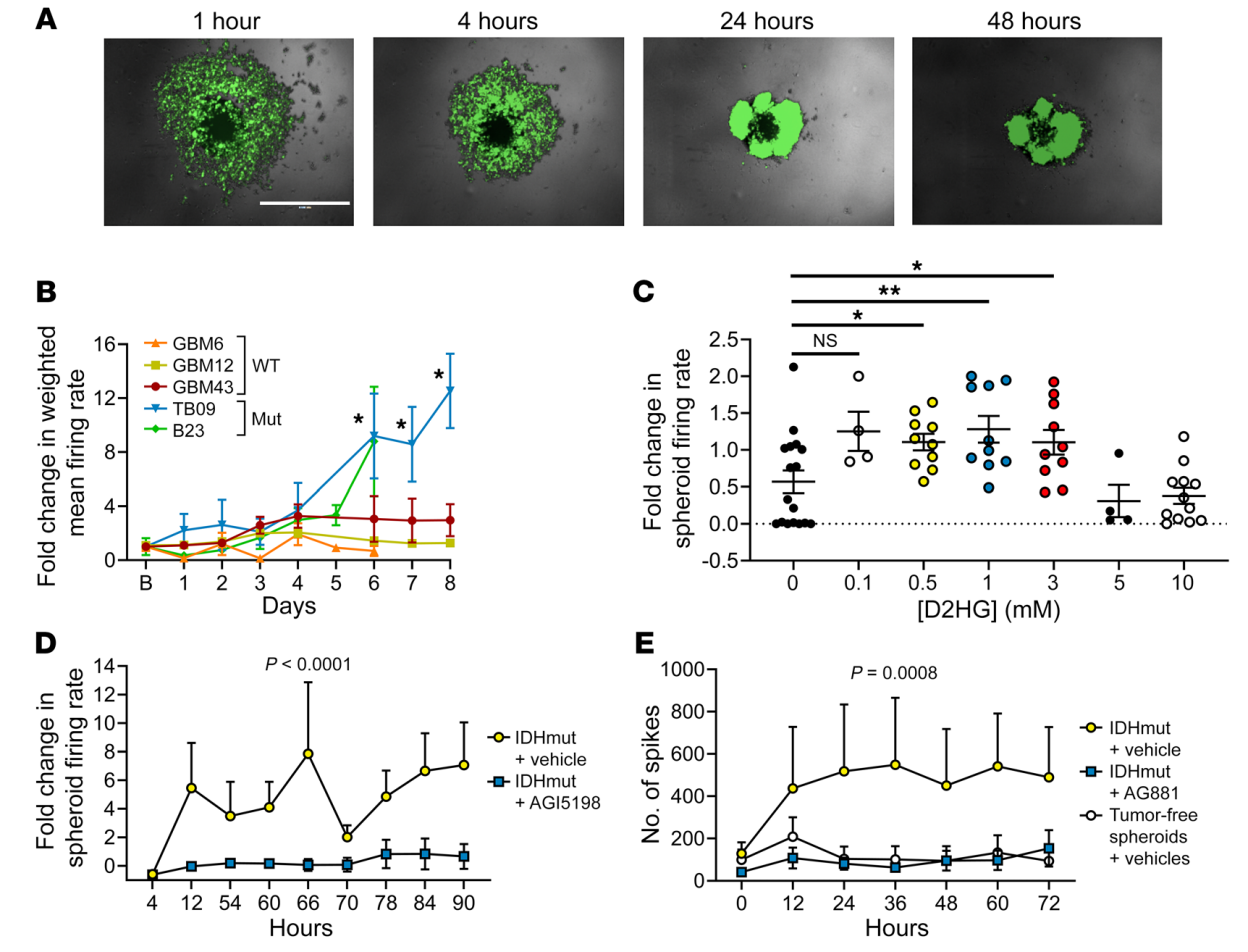
Modeling and treating IDHmut glioma–associated seizure-like activity with cerebral spheroids. (**A**) Serial imaging demonstrates infiltration of GFP-labeled patient-derived TB09 into a spheroid over 1–48 hours. Scale bar: 1 mm. (**B**) Spheroids were cocultured with patient-derived glioma lines (GBM6, *n* = 6; GBM12, *n* = 32; GBM43, *n* = 23; TB09, *n* = 4; B23, *n* = 5), and fold change in weighted mean firing rate was calculated over time. *P < 0.05 vs. IDHwt cells via 2-tailed *t* test. Experiment was repeated twice for a total of 3 times. (**C**) Spheroids were treated with either vehicle (*n* = 16) or 0.1 mM D2HG (*n* = 4), 0.5 mM D2HG (*n* = 10), 1 mM D2HG (*n* = 10), 3 mM D2HG (*n* = 10), 5 mM D2HG (*n* = 4), or 10 mM D2HG (*n* = 12), and fold change of weighted mean firing rate for 10 minutes after treatment was calculated for each well. Bars represent mean ± SEM. *P < 0.05, **P < 0.01 by unpaired, 2-tailed *t* test. Experiment was done in triplicate. (**D**) Spheroids were cultured with patient-derived IDH1mut glioma TB09 and treated with either vehicle (*n* = 6) or 1.5 μM AGI5198 (*n* = 7), and fold change in weighted mean firing rate was calculated over time. Data points represent mean ± SEM. Data were analyzed with 2-way ANOVA. Experiment was done once. (**E**) Spheroids were either cultured alone and treated with vehicle (*n* = 12), or cultured with patient-derived IDH1mut glioma 905 and treated with either vehicle (*n* = 14) or 30 nM AG881 (*n* = 13). Data points represent mean ± SEM. Data were analyzed with 2-way ANOVA. Experiment was done twice.

To further explore the effect of D2HG on neuronal activity, we used mature human induced pluripotent stem cell–derived cortical spheroids composed of both cortical neurons and glial cells in a 3D structure, approximately 500 μm in diameter (31–34). To each spheroid (1 per well in a 96-well plate), we added patient-derived IDHwt (GBM6, GBM12, GBM43) or IDHmut (B23, TB09) glioma cells. Both IDHwt and IDHmut glioma cells rapidly infiltrated the spheroids, such that within hours, cocultures of cortical neurons, glia, and glioma were spontaneously established (Figure 4A, Supplemental Figure 12, and Supplemental Video 1). This behavior comports with what has been previously observed in glioma cells when added to cerebral spheroid/organoid preparations (35–37). While spheroids with admixed IDHwt glioma cells had stable firing rates over time, those with IDHmut glioma cells showed gradually increasing firing rates (*P* < 0.05; Figure 4B). Treatment with 0.5–3 mM exogenous D2HG also caused spheroid firing to increase, even in the absence of glioma cells (Figure 4C). IDHmut glioma–induced spikes in spheroids were reduced by both a first-generation IDH1mut inhibitor, AGI5198 (*P* < 0.0001), and a second-generation IDH1/2 mutant inhibitor, AG881 (*P* = 0.0008) (Figure 4, D and E). IDHmut inhibition had no effect on cultured IDHmut glioma cell number (*P* = 0.72; Supplemental Figure 13A) or viability (*P* = 0.99; Supplemental Figure 13, B and C), but did inhibit the production of 2HG by IDHmut glioma cells (Supplemental Figure 13D).

### Development and characterization of an in vivo model of TAE in IDHwt versus IDHmut glioma.

**Figure 5:**
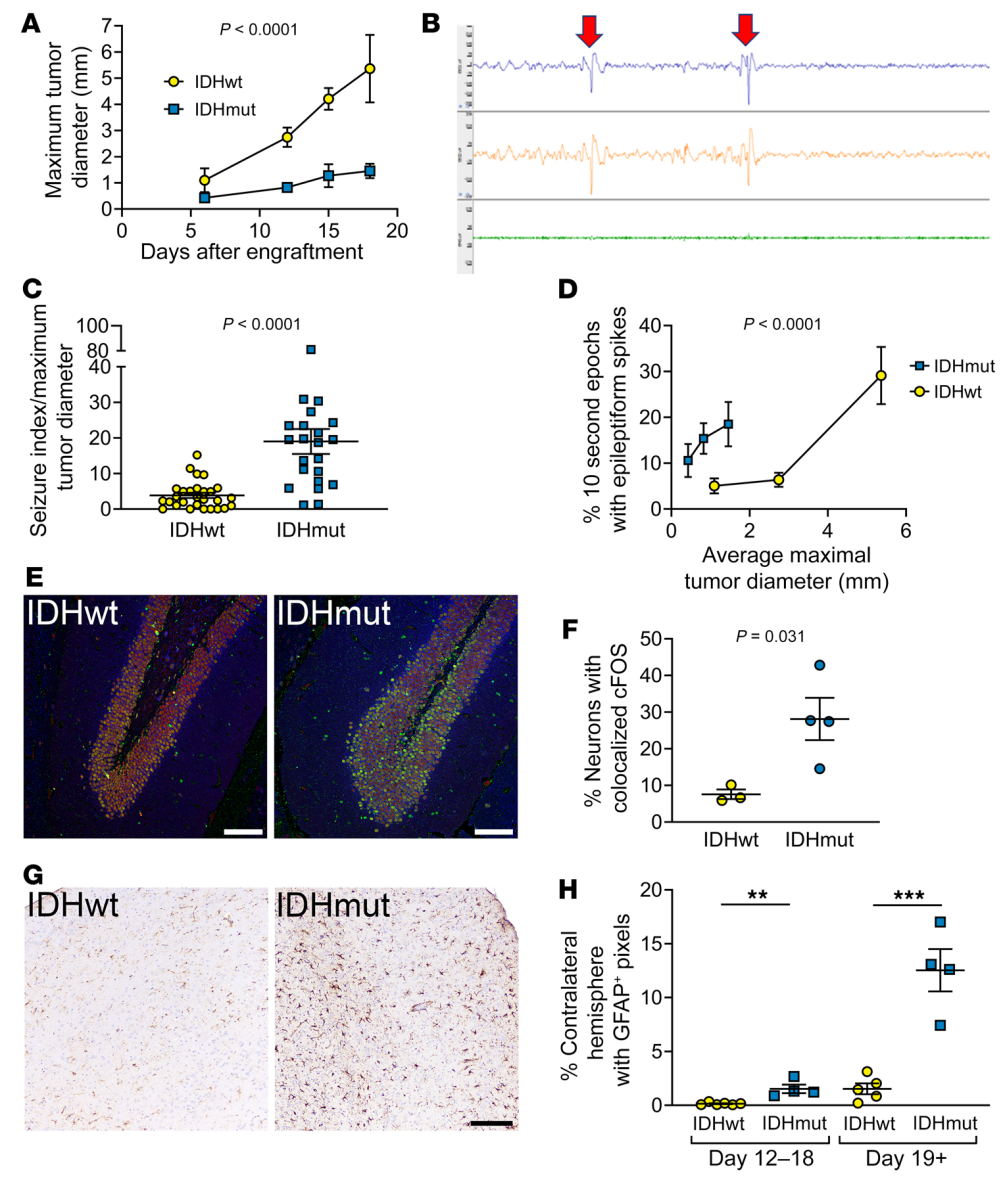
IDHmut glioma causes TAE in mice. (**A**) Mice were engrafted with either IDHmut or IDHwt glioma. Four mice per group were euthanized on days 6, 12, 15, and 18. Intracranial tumor size at each time point was histologically measured as maximum tumor diameter. Data points represent mean ± SEM. Data were analyzed with 2-way ANOVA. (**B**) Top trace is EEG1, middle trace is EEG2, bottom trace is EMG. Red arrows indicate representative epileptiform spike. (**C** and **D**) Mice engrafted with IDHwt (*n* = 12) or IDHmut (*n* = 8) glioma had recordings taken at days 6, 12, and 18. EEGs were scored for percentage of 10-second epochs that contained epileptiform spikes and normalized to the tumor size in IDHwt or IDHmut glioma at day 6, 12, or 18, respectively (**C**), as well as directly compared (**D**). Bars represent mean ± SEM. Data were analyzed with unpaired, 2-tailed *t* test (**C**) or 2-way ANOVA (**D**). (**E**) Dentate gyrus of mice engrafted with either IDHwt or IDHmut glioma, stained with NeuN (1:400; ab104224, red), cFOS (1:500; ab190289, green), and MAP2 (1:1,000; ab5392, blue). (**F**) Percentage of NeuN colocalized with cFOS in the dentate gyrus of mice engrafted with either IDHwt (*n* = 3) or IDHmut (*n* = 4) glioma. Bars represent mean ± SEM. Data were analyzed with unpaired, 2-tailed *t* test. (**G**) Cortex contralateral to IDHwt- or IDHmut-engrafted glioma, stained with GFAP (1:500; CP040A). (**H**) Quantification of GFAP positivity in contralateral cortex of mice engrafted with IDHwt glioma within 12–18 days after engraftment (*n* = 6) or 19+ days after engraftment (*n* = 5) and of mice engrafted with IDHmut glioma within 12–18 days after engraftment (*n* = 4) or 19+ days after engraftment (*n* = 4) using pixel thresholding in QuPath. Scale bars in **E** and **G**: 100 μm. Bars represent mean ± SEM. **P < 0.01, ***P < 0.001 by unpaired, 2-tailed *t* test.

To explore the effect of IDHmut glioma and IDHmut inhibitors on seizure activity in vivo, we developed a model of tumor-associated epilepsy (TAE) based on Sleeping Beauty transposase–engineered IDHwt and IDHmut mouse gliomas provided by Maria Castro (38). Both cell types are driven by mutant NRAS and have inactivation of ATRX and p53 (Supplemental Figure 14A), the latter 2 alterations being a consistent feature of IDHmut astrocytomas (39). Three advantages to this system over most orthotopic patient-derived IDHmut glioma xenograft models are that it has (a) consistent, faster in vivo growth; (b) an immunocompetent microenvironment; and (c) control IDHwt cells that, aside from IDH1 R132H, are isogenic with control IDHmut glioma cells. In this IDHwt-IDHmut mouse glioma pairing, the IDHmut cells expressed the IDH1 R132H protein and produced more 2HG (Supplemental Figure 14, B and C). Mice orthotopically engrafted with IDHwt glioma in their right frontal lobes had shorter median survival than mice with IDHmut glioma (22 vs. 33 days, *P* = 0.0014; Supplemental Figure 14D). While this accurately reflected the less aggressive nature of IDHmut gliomas compared with IDHwt gliomas, as had been previously reported in this model (38), it required controlling for the potentially confounding effects of tumor size and mass effect on seizure activity. In our laboratory, IDHmut gliomas around 15 days after engraftment were of a similar size to IDHwt gliomas at just 6 days after engraftment (Figure 5A). Thus, when seizure activity was recorded via EEG (Supplemental Figure 14, E and F; Figure 5B; and Supplemental Videos 2 and 3), results were evaluated relative to estimated tumor size, based on days after engraftment. Such analyses showed that mice with IDHmut glioma had 4.9-fold more epileptiform spikes than mice engrafted with IDHwt glioma (*P* < 0.0001; Figure 5, C and D). The brains of those mice showed more evidence of seizures distant from the right frontal tumors, as indicated by upregulated cFOS in the hippocampal dentate gyri (Figure 5, E and F) and increased GFAP-positive reactive astrocytosis even in the contralateral cortex (Figure 5, G and H), both of which are sensitive markers of generalized seizures (40, 41). Expression profiling of the peritumoral brain around IDHmut gliomas also showed upregulation of *SLC12A5*, *THBS1*, *VEGFA*, *FOSL2*, and *SYNPO*, as has been previously reported in epileptic brain tissue (Supplemental Figure 15) (42–47). These data show that IDHmut TAE can be effectively modeled in vivo.

### IDHmut inhibitor reduces epileptiform activity of mice engrafted with IDHmut glioma.

**Figure 6:**
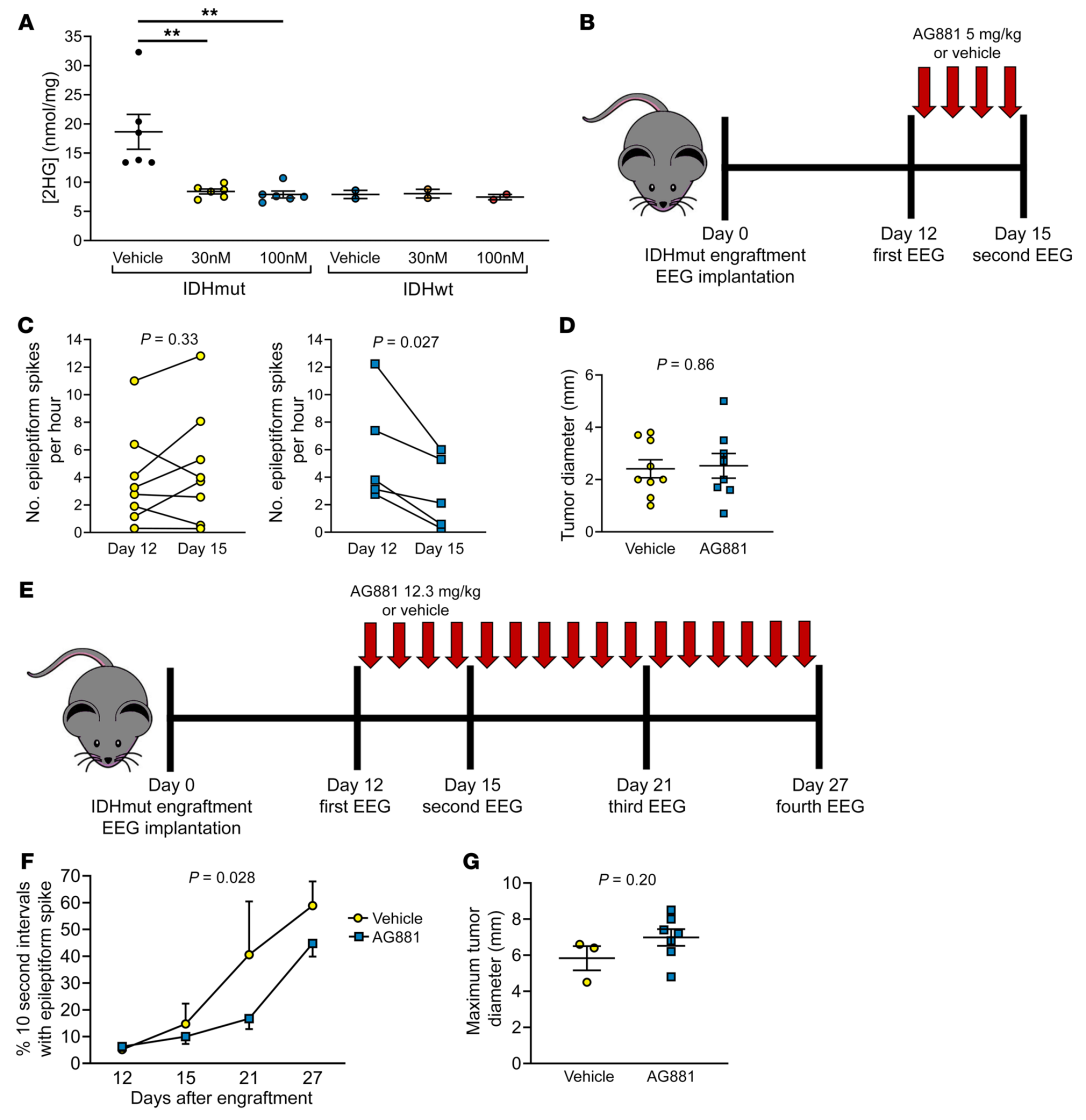
IDHmut inhibitors reduce seizures in IDHmut glioma–engrafted mice. (**A**) IDHmut or IDHwt mouse glioma was treated with vehicle or 30 or 100 nM AG881 for 2 days in vitro. Cell pellets were collected, and intracellular levels of 2HG were assessed with mass spectrometry and normalized to total protein of each cell pellet. IDHmut glioma, *n* = 4 biological replicates; IDHwt glioma, *n* = 2 biological replicates. Bars represent mean ± SEM. **P < 0.01 by unpaired, 2-tailed *t* test (Bonferroni-adjusted *P* = 0.02). (**B**) Schematic of therapeutic study of C57BL/6 mice engrafted with IDHmut glioma and treated daily with 5 mg/kg of AG881 or vehicle. (**C**) EEGs of mice engrafted with IDHmut glioma and treated with vehicle (*n* = 8) or 5 mg/kg AG881 (*n* = 5) were blinded to treatment status and time point, and number of epileptiform spikes per hour was counted at baseline and at day 15. Data points represent each mouse, and lines connect the paired EEGs for a given mouse from different time points. Data were analyzed with paired, 2-tailed *t* test. (**D**) Maximal tumor diameter was measured from H&E-stained brains engrafted with IDHmut glioma treated with vehicle (*n* = 8) or 5 mg/kg AG881 (*n* = 5). Bars represent mean ± SEM. Data were analyzed with unpaired, 2-tailed *t* test. (**E**) Schematic of therapeutic study of C57BL/6 mice engrafted with IDHmut glioma and treated daily with 12.3 mg/kg of AG881 or vehicle. (**F**) EEGs of mice engrafted with IDHmut glioma and treated with vehicle (*n* = 3) or 12.3 mg/kg AG881 (*n* = 7) were blinded to treatment status and time point, and scored for percentage of 10-second epochs that contain epileptiform spikes. Data points represent mean ± SEM. Data were analyzed with 2-way ANOVA. (**G**) Maximal tumor diameter was measured from H&E-stained brains engrafted with IDHmut glioma treated with vehicle (*n* = 3) or 12.3 mg/kg AG881 (*n* = 7). Data were analyzed with unpaired, 2-tailed *t* test.

We next determined whether AG881 could reduce seizure activity. In vitro, 30 nM and 100 nM AG881 lowered production of 2HG by the IDHmut cells to that of the IDHwt mouse glioma (Figure 6A) but did not affect cell viability (Supplemental Figure 13C). In an initial experiment, 5 mg/kg daily of AG881 reduced epileptiform spikes in IDHmut-engrafted mice by 51% (*P* = 0.027; Figure 6, B and C) without any effect on tumor size (Figure 6D). Next, 12.3 mg/kg daily of AG881, which more closely corresponds to the oral daily dose given to human patients (48), was used over a longer time frame (Figure 6E). As before, AG881 reduced epileptiform spikes in IDHmut-engrafted mice compared with vehicle over the course of the entire 15-day recording period (*P* = 0.028; Figure 6F), again with no effect on tumor size (Figure 6G). (In the latter experiment, by day 27 IDHmut tumors had reached a size at which mass effect likely contributed to seizure activity independent of D2HG suppression.) Thus, clinical IDHmut inhibition can reduce seizures, apart from any effects on tumor growth.

## Discussion

Prior to the molecular era of brain tumor research it had been known that, among patients with diffusely infiltrative gliomas, seizures were more likely in younger adults and in WHO grade 2 to 3 gliomas (49). We and others showed that this was largely due to IDHmut being more common in younger patients and in WHO grade 2 to 3 gliomas (1, 21–23). In the current study, we found that postoperative risk of seizures in patients varies greatly depending on several factors (including glioma genotype), that the D2HG oncometabolite produced by IDHmut increases neuronal excitation in a glia-dependent manner, and that an IDHmut inhibitor currently being explored in clinical trials reduces epileptiform activity in IDHmut-engrafted mice independent of effects on tumor growth.

A key requirement of any clinical trial associated with seizure reduction by IDHmut inhibitors is knowing which patients are at highest risk, and which patients would therefore likely benefit the most. Previous work by others demonstrated that the extent of surgical resection and incidence of preoperative seizures are important risk factors of postoperative seizures in low-grade glioma patients (9, 50, 51). More recent work showed that IDHmut was associated with increased seizures during the post-surgical disease course (52). The current study extended those findings by using precise time-to-event analyses over longer follow-up intervals, and by incorporating the most updated WHO molecular-based classification system of adult-type diffuse gliomas. Our data showed that, while both IDHmut astrocytomas and oligodendrogliomas had similarly high rates of seizures before the first surgery, those two IDHmut glioma subtypes diverged markedly after clinical intervention, such that IDHmut astrocytoma patients developed postoperative seizures fastest, IDHmut oligodendroglioma patients were the slowest, and IDHwt glioblastoma patients were in between. This suggests that several factors contribute to the risk of postoperative seizure once the natural history of the disease has been altered, including whether a glioma is IDHmut, the aggressiveness of glioma regrowth, glioma location, and the extent to which it was initially resected. For example, even though all oligodendrogliomas have IDHmut, they are more responsive to treatment with radiation therapy and/or chemotherapy and regrow and infiltrate the brain at a much slower rate than IDHmut astrocytomas. Although IDHmut astrocytomas are less aggressive than IDHwt glioblastomas, IDHmut astrocytomas produce and release D2HG whereas IDHwt glioblastomas do not.

Judging when a glioma has clinically recurred, and thus when a second-line therapy should be initiated, is often challenging. Seizures have been proposed as a surrogate marker for glioma response to therapy (53), and seizures often precede glioma progression (50, 54–56). Our study extends these observations in a large cohort of molecularly defined adult-type diffuse gliomas, demonstrating that postoperative seizures often arise shortly before IDHwt glioblastoma recurrence, particularly when those seizures either occur 6 months or longer after surgical resection and/or worsen in quality. This may represent a vicious-cycle phenomenon of neuronal hyperexcitability and glioma progression (57), as even IDHwt glioblastomas can sometimes trigger neuronal activity (58, 59). In IDHmut astrocytomas and IDHmut oligodendrogliomas, postoperative seizures may occur when tumors have regrown enough to release sufficient D2HG around admixed neurons and glial cells. As those latter sets of gliomas recur and progress more slowly than IDHwt glioblastomas, their cutoffs for seizures as indicators of recurrence/progression are likely to be longer than the 6 months we established for IDHwt glioblastomas. Such IDHmut-themed studies are ongoing.

While IDHmut clearly affects pre- and postoperative seizure risk, the mechanism(s) by which IDHmut gliomas trigger seizures has been more difficult to establish. Within the tumor cell, D2HG is well known to inhibit certain histone and DNA demethylases that require α-ketoglutarate as a cosubstrate. Thus, IDHmut gliomas have increased histone and DNA methylation (60, 61). Less is known about D2HG effects outside the glioma cell, even though D2HG is exported into the microenvironment (25). Here we showed, for the first time to our knowledge, that the pro-excitatory effects of D2HG on neurons are rapid and depend on the presence of non-neoplastic glial cells, that such effects do not appear to be directly through ionotropic glutamate receptors, that D2HG is unlikely to be blocking the reuptake of glutamate by astrocytes near the synaptic cleft, and that the mTOR pathway may not be involved. There appears to be a biphasic firing pattern immediately following the application of D2HG in the neuron-glia cocultures. This biphasic pattern includes an initial rapid firing pattern for the first minute or two of treatment, followed by a slower, more rhythmic bursting firing pattern. It is still unclear whether D2HG acts directly on glial cells (mostly astrocytes) rather than on neurons to trigger release of glial neurotransmitter stores or other excitatory compounds, or whether glial cells somehow “prime” neurons to respond to D2HG, especially as concerns the delayed rhythmic pattern of bursting. Such research is ongoing.

Nevertheless, it is clear that IDHmut glioma cells trigger neuronal excitation through D2HG release, as evidenced by how D2HG was sufficient to trigger network bursts in vitro, and how both first-generation (AGI5198) and second-generation (AG881) IDHmut enzyme inhibitors blocked the pro-excitatory effects of IDHmut glioma cells in spheroid cultures. In particular, the brain-penetrant AG881 (vorasidenib) inhibited neuronal firing in both IDHmut glioma–spheroid cocultures and mice engrafted with IDHmut gliomas. In a phase I trial of IDHmut glioma patients, AG881 reduced 2HG levels within gliomas by 95%, was well tolerated, and had favorable pharmacokinetics (26). Consistent with this, we found that AG881 profoundly inhibited 2HG production and seizure activity. In our immunocompetent in vivo model of TAE, based on previously published mouse glioma cells that have ATRX and TP53 inactivation and are isogenic other than the presence or absence of IDHmut (38, 62), the IDHmut is only a passenger mutation, yet still produces D2HG. Thus, we were able to show that IDHmut inhibition could help control TAE, independent of any other antitumor effects that clinical trials might ultimately reveal. This aligns with isolated clinical reports and small case series suggesting that IDHmut inhibitors substantially and rapidly reduce seizures, even when those seizures are resistant to multiple ASMs (63–65). The current data could therefore inform a clinical trial of IDHmut inhibitor with seizure control as a primary endpoint, especially since reducing seizures is becoming a higher priority in glioma clinical trials (53).

In summary, our data suggest the following: (a) risk factors for postoperative seizures in adult-type diffuse glioma patients include glioma molecular subtype, preoperative seizures, tumor location, and extent of resection; (b) IDHmut-associated seizures can be experimentally recapitulated in vitro and in vivo; (c) the pro-excitatory effects of IDHmut can be attributed to its D2HG product; (d) D2HG rapidly triggers neuronal firing in a glia-dependent manner; and (e) IDHmut inhibitors may help mitigate TAE in IDHmut glioma patients. These findings advance our understanding of how IDHmut gliomas affect the brain, and provide a rationale for a clinical trial specifically aimed at using IDHmut inhibitors to improve seizure control.

## Methods

### Cell culture, Western blotting, and 2HG liquid chromatography–mass spectrometry.

For non-neoplastic neuronal and neuronal-glial cultures, P0 postnatal mice (The Jackson Laboratory, strain 000664) were euthanized, and the brain was removed. Cortical tissue was isolated, digested with Accutase and mechanical pipetting, and passed through a 70 μm strainer. Cells were plated on cell culture–treated plates that were coated with poly-d-lysine (R&D Systems, 3439-100-01) in base medium composed of Neurobasal A Medium (Gibco, 10888-022), 2% B-27 (Gibco, 17504044), 2 mM l-glutamine (Corning, 25005-Cl), and 0.5% penicillin-streptomycin (Corning, 3000-Cl). On day 1 of cell culture, cells were fed with half change of base medium supplemented with 100 mM glucose. To remove glial cells, and establish postmitotic neuron-only cultures, on day 1 of cell culture, cells were fed with half change of base medium supplemented with 100 mM glucose and 2 μM Ara-C (Sigma-Aldrich, C6645) in order to achieve 1 M Ara-C in the complete medium. Cells were fed every 3–4 days with half change of medium supplemented with 100 mM glucose.

IDHmut and IDHwt mouse–derived and patient-derived glioma cells were grown in medium composed of 50% Neurobasal A medium and 50% DMEM/F12 medium supplemented with 1× B-27, 1× N-2, 1× nonessential amino acids, 20 ng/mL EGF, 20 ng/mL bFGF, 0.5 mM l-glutamine, 0.0002% heparin, and 100 U/mL penicillin-streptomycin. IDHmut and IDHwt mouse gliomas were shared by Maria Castro’s laboratory (University of Michigan, Ann Arbor, Michigan, USA). TB09 cells were shared by Timothy Chan’s laboratory (Cleveland Clinic, Cleveland, Ohio, USA). B23 cells were shared by Albert Kim’s laboratory (Washington University in St. Louis, St. Louis, Missouri, USA). GBM6, GBM12, and GBM43 cells were obtained from the Mayo Clinic Xenograft National Resource. Cells were maintained in a humidified incubator with 5% CO_2_ and at 37°C. All cell lines were authenticated by short tandem repeat sequencing.

For Western blotting, cells were lysed with RIPA buffer containing phosphatase/protease inhibitor cocktail, and protein concentration of cellular lysates was quantified using a Bradford assay. Proteins were loaded in a 4%–12% Bis-Tris polyacrylamide gel and transferred to a PVDF membrane. Antibodies included anti–IDH1 R132H (Dianova, DIA-H09; 1:250), anti-GAPDH (Cell Signaling, D16H11, 5174; 1:1,000), anti–phospho-S6 (Cell Signaling, Ser235/236, 2211; 1:1,000), and anti–β-actin (Cell Signaling, 8H10D10, 3700; 1:1,000) primary antibodies and HRP-conjugated secondary antibodies (Cell Signaling, 7074 and 7076; 1:10,000).

To assess intracellular 2HG, extraction buffer composed of 80% methanol and 20% H_2_O cooled to –80°C was added to cell pellets, and to assess extracellular 2HG, 800 μL of methanol cooled to –80°C was combined with 200 μL of conditioned medium. Eight micromolar ^13^C5 d-2 hydroxyglutarate was included in the extraction buffer as an internal control. Samples were incubated at –80°C for 5 minutes, then brought to room temperature and then vortexed for 1 minute; this process was repeated twice for a total of 3 times. After storage at –80°C overnight, samples were centrifuged at 16,000*g* for 15 minutes at 4°C. The supernatant was submitted for liquid chromatography–tandem mass spectrometry, and the *m*/*z* transition used was 147 → 129. The precipitate was sonicated at 50% amplitude for 2 minutes in RIPA buffer, and then protein was quantified with a Bradford assay.

### Formation of human induced pluripotent stem cell–derived 3D cortical neuron–astrocyte cocultures.

Preplated 3D human cortical neural spheroidal cocultures were provided by StemoniX Inc. in the form of the StemoniX MicroBrain 3D Assay Ready product (31–34). Each well of the plate contained a single, free-floating human induced pluripotent stem cell (iPSC)–derived cortical 3D neural cell culture generated from neural progenitor cells obtained from a single human donor source. These spheroids contain astrocytes as well as neurons, and show synaptophysin-positive puncta outlining MAP2-positive neurites (31, 33, 34, 66). They are also electrophysiologically mature, with synchronized spontaneous activity (31, 32, 34).

StemoniX shipped the MicroBrain 3D Assay Ready plates overnight at ambient conditions. Upon receipt, plates were processed according to detailed instructions provided. Briefly, after unpacking, the plates were centrifuged for 2 minutes at 200*g* (Sorvall centrifuge) and then inspected using a light microscope to ensure the presence of a single spheroid in each well. Plate exteriors were decontaminated with 70% ethanol. After unsealing, the medium was changed (half of the total volume, 3 times) using BrainPhys Neuronal Medium SM1 Kit (STEMCELL Technologies, 05792) supplemented with 20 ng/mL of recombinant human BDNF (STEMCELL Technologies, 78005), 20 ng/mL of GDNF (STEMCELL Technologies, 78058), and 1× penicillin-streptomycin (GE Healthcare Life Sciences) or the NeuralX Cortical Neuron media kit (StemoniX, NXCNM-AA-0250). Spheroids were maintained at 37°C and 5% CO_2_ until used in experiments. Half medium changes were performed every Monday, Wednesday, and Friday using 1 of the 2 media described above, which are interchangeable according to the manufacturer’s instructions.

### Multi-electrode array analyses and patch clamping.

Forty-eight-well multi-electrode array plates (Axion Biosystems, M768-tMEA-48W) were coated with poly-d-lysine (R&D systems, 3439-100-01). Cortical lysates were plated at 50,000 cells per 10 μL droplet on top of the 4 × 4 grid of 16 electrodes and allowed to adhere for 45 minutes, and then 490 μL base medium was added. On day 21, baseline recordings were taken for 5 minutes, and treatment recordings were taken for at least 5 minutes. Treatments included either vehicle, D2HG (Sigma-Aldrich, H8378), or dihydrokainic acid (Abcam, ab120066). For spheroid experiments, spheroids were cultured in Axion plates (M768-tMEA-96W). Spheroids were attached with 3D Matrigel (hESC-Qualified Matrix, Corning, 354277) by placement of a 5 μL dot in the center of the electrode array. Baseline recordings were taken for 5 minutes, and treatment recordings were taken for 5 minutes. A commercial system (Maestro Pro, Axion Biosystems) and its companion software (Axion Biosystems Integrated Studio, AxIS) served as the basis for collection and, in some cases, analysis of electrophysiological data. The system recorded from all electrodes simultaneously with a sampling rate of 12.5 kHz, with real-time display capabilities. Data collection used the neural spikes setting, with a gain of 1,000× and a bandpass filter between 200 Hz and 4 kHz. The AxIS software labels spikes as signals with amplitudes above a threshold of 6 standard deviations from the average noise level, bursts as a minimum of 5 spikes with a maximum interspike interval of 100 ms, and network bursts as a minimum of 50 spikes from at least 35% of the electrodes with a maximum interspike interval of 100 ms. For glioma-spheroid coculture experiments, 10,000 glioma cells were added to wells containing spheroids.

Homomeric GluA4(flip) AMPA receptors, GluK2 kainate receptors, GluN1/2A NMDA receptors, GluN1/2B NMDA receptors, or excitatory amino acid isoform GLT1a were transiently expressed in HEK293 cells, and whole-cell patch clamp recordings were performed.

### Intracranial murine glioma TAE model.

Six- to eight-week-old female C57BL/6 mice were purchased from The Jackson Laboratory (strain 000664). Mice were monitored daily for neurological symptoms of brain tumors, including hunched posture, altered gait, lethargy, and decreased body weight.

Intracranial tumors were established in C57BL/6 mice by injection of 50,000 glioma cells in 2 μL PBS over 1 minute using a Hamilton syringe into the right cortex/caudate/putamen 0.5 mm anterior, 2 mm lateral, and 2.5 mm deep relative to the bregma. EEG/electromyograph (EMG) headmount (Pinnacle Technology, 8201) was implanted by first gluing the headmount to the skull such that the front edge of the headmount was 3 mm anterior of the bregma. Next, screws (Pinnacle Technology, 8209) were placed at each corner of the headmount, silver epoxy (Pinnacle Technology, 8226) was applied on the underside and outer corner of each screw, and then the screws were screwed into the skull. The EMG wires were placed into the nuchal muscles, and then dental acrylic was applied to the headmount base before the incision was sutured closed.

Mice were allowed to recover for several days after tumor engraftment and EEG device implantation before EEG recordings. Mice were placed in a large mouse cage (Pinnacle Technology, 8228) with food and water. A preamplifier (Pinnacle Technology, 8202) was connected to the EEG headmount and to the commutator/swivel (Pinnacle Technology, 8204), which was connected to the 3-channel analog adapter (Pinnacle Technology, 8242), which was connected to a computer. EEG recordings with video were acquired using the software Sirenia Acquisition (Pinnacle Technology) for up to 8 hours. EEG recordings were exported, and blinded to tumor genotype, treatment condition, and time point after tumor engraftment. EEGs were reviewed for epileptiform spikes by a board-certified neurologist. Because of the high incidence of spikes in IDHmut-engrafted mice, instead of counting of the number of individual spikes per recording, the percentage of 10-second intervals containing epileptiform spikes was quantified.

AG881 was prepared daily, within 1 hour of in vivo administration. AG881 was suspended in 0.5% methylcellulose (cP 400) and 0.2% Tween-80 in H_2_O, vortexed for 2 minutes, and then further suspended with low sonication. AG881 or vehicle was administered once after baseline EEG recording and then thereafter every morning. Mice were randomized to treatment group.

For peritumoral expression profiling, mice were euthanized on day 12 after tumor engraftment and the brain removed. Tumor was removed from the brain via microdissection, and then the peritumoral tissue was quickly removed and placed in RNAlater solution (Invitrogen, AM7021). RNA was extracted from brain tissue using RNeasy Mini Kit (Qiagen, 74104). mRNA libraries were constructed, and differentially expressed genes were compared across IDHmut and IDHwt groups.

### Immunohistochemistry and immunocytochemistry.

Formalin-fixed paraffin-embedded tissue was sectioned at a thickness of 4 μm, and deparaffinization and hydration were performed with xylene and ethanol, followed by antigen retrieval, washing, and blocking with 10% goat serum in TBS. Frozen spheroids were sectioned at a thickness of 10 μm. For immunocytochemistry, cells were fixed in 4% paraformaldehyde, and then washed in PBS. Primary antibody was incubated overnight, and then either secondary immunofluorescent antibodies or HRP-conjugated antibodies were incubated for 1 hour. Primary antibodies used were as follows: NeuN (Abcam, 1B7, ab104224; 1:400), cFOS (Abcam, ab190289; 1:500), MAP2 (Abcam, ab5392; 1:1,000), GFAP (IHC: Biocare, CP040A, 1:500; immunofluorescent: Abcam, ab5804, 1:1,000), phospho-S6 (Cell Signaling, Ser235/236, 2211; 1:1,000), synaptophysin (Thermo Fisher Scientific, SY38, MA1-213; 1:200), PSD95 (Abcam, ab18258; 1:200), ALDH1L1 (Abcam, ab177463; 1:500). Secondary immunofluorescent antibodies were used at 1:250 dilution and included A11031 (Thermo Fisher Scientific), 111-545-144 (Jackson ImmunoResearch Laboratories), ab175477 (Abcam), and ab150171 (Abcam). Samples were coverslipped with mounting medium with DAPI (Abcam, ab104139). Slides were imaged on a Nikon Eclipse Ni-E or Leica DMi8 microscope. For immunocytochemistry of intact 3D spheroids, primary antibodies (GFAP, Agilent, Z033429-2, 1:1,000; Neurofilament, BioLegend, SMI-312, 1:1,000) were incubated overnight in 10% donkey serum, and then incubated in secondary immunofluorescent antibodies (Thermo Fisher Scientific, A-21206 and A-31571; 1:1,000) for 1 hour. For colocalization studies, slides were imaged and ND2 files were analyzed in ImageJ (NIH) via colocalization color thresholding. For counting of synaptic puncta, slides were analyzed in ImageJ with the SynapseCounter plug-in. For quantitative analysis of immunohistochemistry stains, slides were first digitized, and then analyzed in QuPath (0.3.2) (67). Pixel thresholding classifiers were established with positive and negative control tissue stains, and then applied to immunohistochemistry stains with non-nuclear staining to determine number of positive pixels per total number of pixels per image.

### Clinical chart review.

The cohort consisted of 247 consecutive patients with WHO grade 2–4 adult-type diffuse gliomas who underwent first surgical resection between 2014 and 2019 and who had at least 1 follow-up visit. Patients were excluded if they did not have their first surgical resection at Northwestern Medicine hospitals, because detailed data from outside hospitals on their pre-surgical, peri-surgical, and post-surgical events were limited. Seizure frequency and symptoms were identified through review of patients’ medical charts. Postoperative seizure was defined as any seizure at least 7 days after initial surgical resection. Extent of surgical resection and localization of tumor were defined through review of pre-surgical and post-surgical MRIs by the medical care team of each patient. In comparisons of gross total resection with subtotal resection, near-total resection (defined as at least 90% tumor resected) was included in the gross total resection category. Tumor progression in this retrospective study was defined by the patient’s medical care team. Instead of retrospective definition of when each patient demonstrated tumor progression on their MRIs, a patient was deemed to have tumor recurrence/progression if they (a) underwent and/or were recommended to undergo second-line therapy for tumor recurrence/progression by their medical care team and/or (b) experienced definite clinical deterioration not attributable to any cause other than tumor changes, which was also defined by their medical care team at that time.

The quality of preoperative and postoperative seizures was defined in this study by first documenting all symptoms that a patient experienced with their seizures. Next, the seizure status of patients was categorized into groups based on their symptoms: aura, focal seizure, generalized convulsions, or no preoperative seizure. The categories of preoperative versus postoperative seizures were then compared in a tiered order, as generalized convulsions were deemed worse than focal seizures which were deemed worse than auras. A patient was deemed as having a worse seizure if any of the following were true: (a) their postoperative seizure was qualitatively worse than their preoperative seizure; (b) they never had a preoperative seizure and then had a seizure for the first time postoperatively; (c) their postoperative seizure was of the same category as their preoperative seizures, but they experienced a cluster of that seizure type. Eight patients had a first postoperative seizure that was not worse than their preoperative seizures, but subsequently did have a worsened postoperative seizure, and in subsequent analyses of how quality of seizures related to timing of tumor progression, the date of their worsened seizure was used. In addition, 7 patients had seizures soon after initiation of second-line therapy (re-resection, *n* = 4; chemotherapy, *n* = 3) in response to tumor progression. Therefore, we compared time to tumor progression with time of postoperative seizure in the 61 patients who did not have seizures associated with second-line therapy. This is because our primary analyses were to understand the co-occurrence of tumor progression and seizures. Including these 7 patients in our analyses would have incorrectly strengthened the association of tumor progression and seizures, since the seizures happened shortly after the start of second-line therapy, which in turn happened shortly after tumor progression.

### Data availability.

RNA-Seq data were deposited in the NCBI’s Gene Expression Omnibus database (GEO GSE227995).

### Statistics.

Statistical significance of differences between groups was determined via 2-sample, 2-tailed *t* test, 1-way ANOVA, Fisher’s exact test, χ^2^ test, or log-rank test as appropriate using GraphPad Prism software. To determine the association between predictors and postoperative seizures, univariable and multivariable analyses were conducted within the framework of a Cox proportional hazards model adjusting for predictors as additive effects, and hazard ratios and corresponding 95% confidence intervals were calculated using R (4.0.2). Unless otherwise specified, data represent mean ± SEM. In all analyses, *P* less than 0.05 was considered significant.

### Study approval.

Deidentified patient data were collected through detailed chart review via a protocol approved by the Northwestern University Feinberg School of Medicine Institutional Review Board, and in accordance with the Declaration of Helsinki. All mice were housed in a barrier facility and handled in compliance with the Institutional Animal Care and Utilization Committee of Northwestern University, which approved the study.

## Author contributions

MRD, WW, TKS, RJ, KYC, BW, KB, DU, VT, JW, AS, KM, JJP, EM, LSS, JK, and JWT obtained the clinical and experimental data. KBB analyzed the clinical data. MRD and CH wrote the manuscript. RVL, JDF, ABH, CKF, and GTS provided manuscript edits. CH conceived of the project and, together with GTS, secured funding.

## Supplementary Material

Supplemental data

Supplemental video 1

Supplemental video 2

Supplemental video 3

## Figures and Tables

**Table 1:**
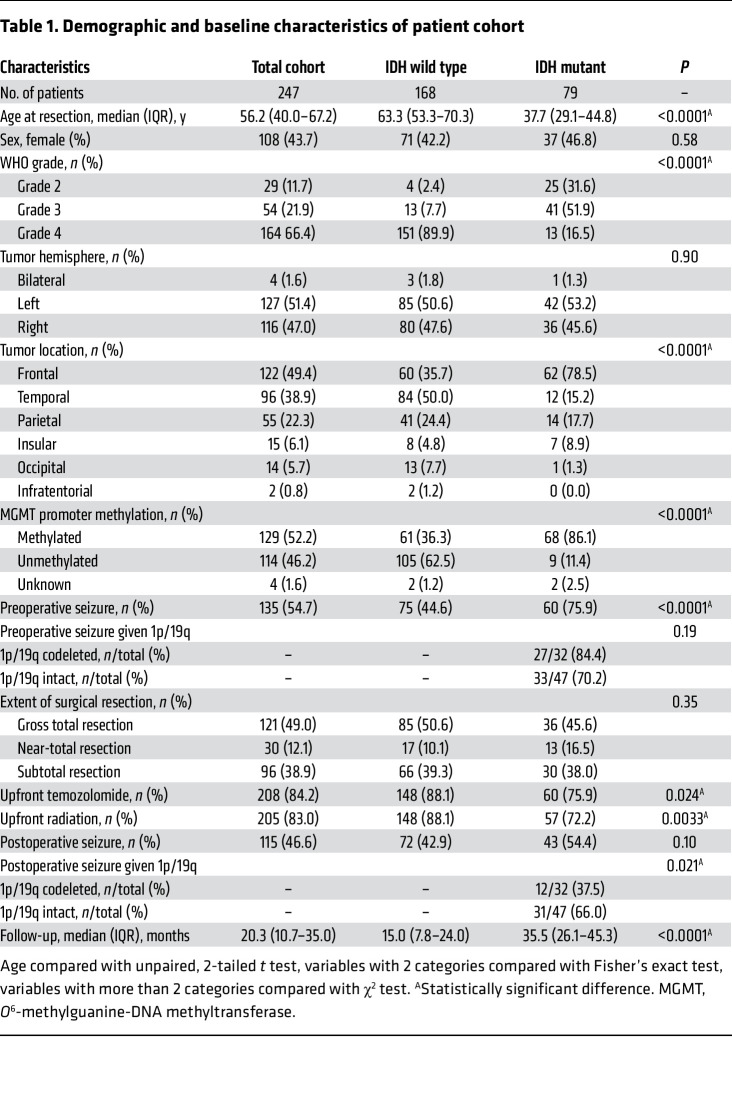
Demographic and baseline characteristics of patient cohort

**Table 2:**
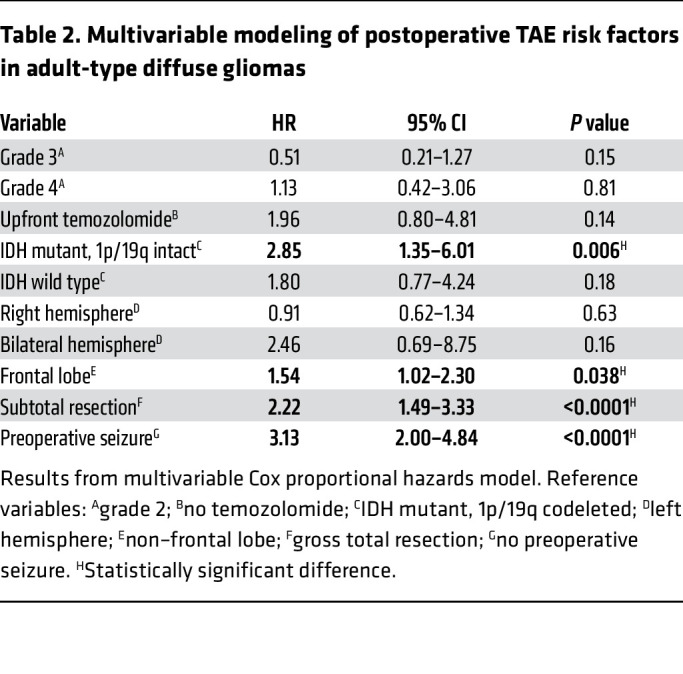
Multivariable modeling of postoperative TAE risk factors in adult-type diffuse gliomas

## Version 1 04/27/2023

In-Press Preview

## Version 2 06/15/2023

Electronic publication
