# Supplementary material for: Postoperative risk of IDH-mutant glioma–associated seizures and their potential management with IDH-mutant inhibitors
Source: J Clin Invest. 2023 Jun 15;133(12):e168035. doi: 10.1172/JCI168035 (PMC10266777; doi:10.1172/JCI168035)
Supplement: Supplemental data [file jci-133-168035-s185.pdf]

Supplemental data for

Postoperative risk of IDH mutant glioma-associated seizures and their potential management with IDH mutant inhibitors

Michael R. Drumm,[1] Wenxia Wang,[1] Thomas K. Sears,[1] Kirsten Bell-Burdett,[2] Rodrigo Javier,[3] Kristen Y. Cotton,[4] Brynna Webb,[5] Kayla Byrne,[6] Dusten Unruh,[7] Vineeth Thirunavu,[1] Jordain Walshon,[1] Alicia Steffens,[1] Kathleen McCortney,[1] Rimas V. Lukas,[8,9] Joanna J. Phillips,[10] Esraa Mohamed,[10] John D. Finan,[11] Lucas Santana-Santos,[12] Amy B. Heimberger,[1,9] Colin K. Franz,[8,13,14] Jonathan Kurz,[15] Jessica W. Templer,[8,9] Geoffrey T. Swanson,[5] Craig Horbinski[1,9,12]

[1]Department of Neurological Surgery, Northwestern University, Chicago, IL, USA
[2]Department of Preventive Medicine, Northwestern University, Chicago, IL, USA
[3]University of Chicago Pritzker School of Medicine, Chicago, IL, USA
[4]University of Illinois at Chicago, Chicago, IL, USA
[5]Department of Pharmacology, Northwestern University, Chicago, IL, USA
[6]Northwestern University, Evanston, IL, USA
[7]Bio-Techne, Minneapolis, MN, USA
[8]Ken & Ruth Davee Department of Neurology, Northwestern University, Chicago, IL, USA
[9]Lou and Jean Malnati Brain Tumor Institute of the Robert H. Lurie Comprehensive Cancer Center, Northwestern University, Chicago, IL, USA
[10]Department of Neurological Surgery, Brain Tumor Center, University of California, San Francisco, San Francisco, CA
[11]Department of Mechanical and Industrial Engineering, University of Illinois at Chicago, Chicago, IL, USA
[12]Department of Pathology, Northwestern University, Chicago, IL, USA
[13]Department of Physical Medicine and Rehabilitation, Northwestern University, Chicago, IL, USA
[14]Biologics Laboratory, Shirley Ryan AbilityLab, Chicago, IL, USA
[15]Merck & Co., Inc., North Wales, PA, USA

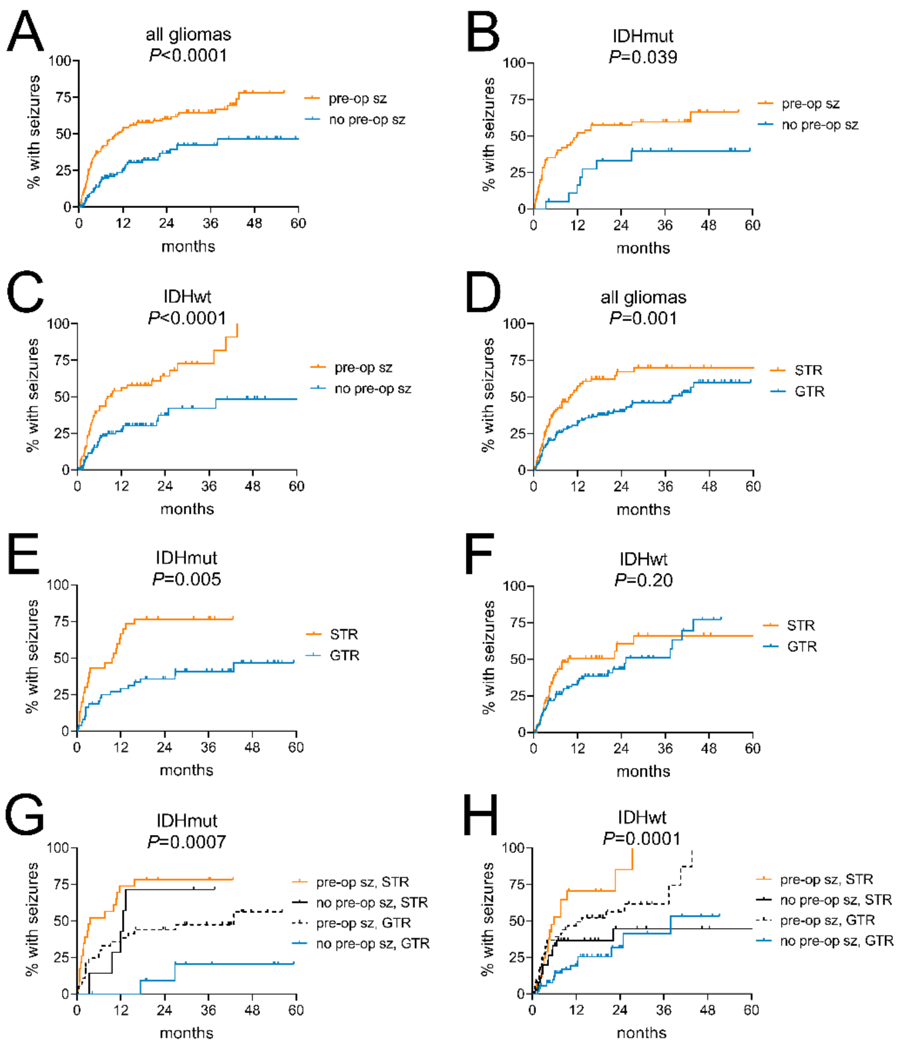

Supplemental Figure S1: Parameter associated with postoperative seizure risk in IDHwt and IDHmut gliomas. (**A-H**) Time-to-event analyses performed for postoperative seizures in patients with diffusely infiltrative glioma, stratified by whether patients experienced pre-operative seizures across (**A**) all patients (total n=247; n=112 with no pre-operative seizure, n=135 with pre-operative seizure), (**B**) patients with IDHmut glioma (total n=79; n=19 with no pre-operative seizure, n=60 with pre-operative seizure), (**C**) patients with IDHwt glioma (total n=168, n=93 with no pre-operative seizure, n=75 with pre-operative seizure), or stratified by extent of surgical resection across (**D**) all patients (total n=247; n=151 with gross total resection, n=96 with subtotal resection), (**E**) patients with IDHmut glioma (total n=79; n=49 with gross total resection, n=30 with subtotal resection), (**F**) patients with IDHwt glioma (total n=168; n=102 with gross total resection, n=66 with subtotal resection), or stratified by both pre-operative seizures and extent of surgical resection across (**G**) patients with IDHmut glioma (total n=79; n=12 no pre-operative seizure and gross total resection, n=7 no pre-operative seizure and subtotal resection, n=37 pre-operative seizure and gross total resection, n=23 pre-operative seizure and sub-total resection) or (**H**) patients with IDHwt glioma (total n=168; n=53 no pre-operative seizure and gross total resection, n=40 no pre-operative seizure and subtotal resection, n=49 pre-operative seizure and gross total resection, n=17 pre-operative seizure and subtotal resection. Data were analyzed with the log-rank (Mantel-Cox) test. Abbreviations: GTR = gross total resection, STR = subtotal resection, pre-op sz = pre-operative seizure.

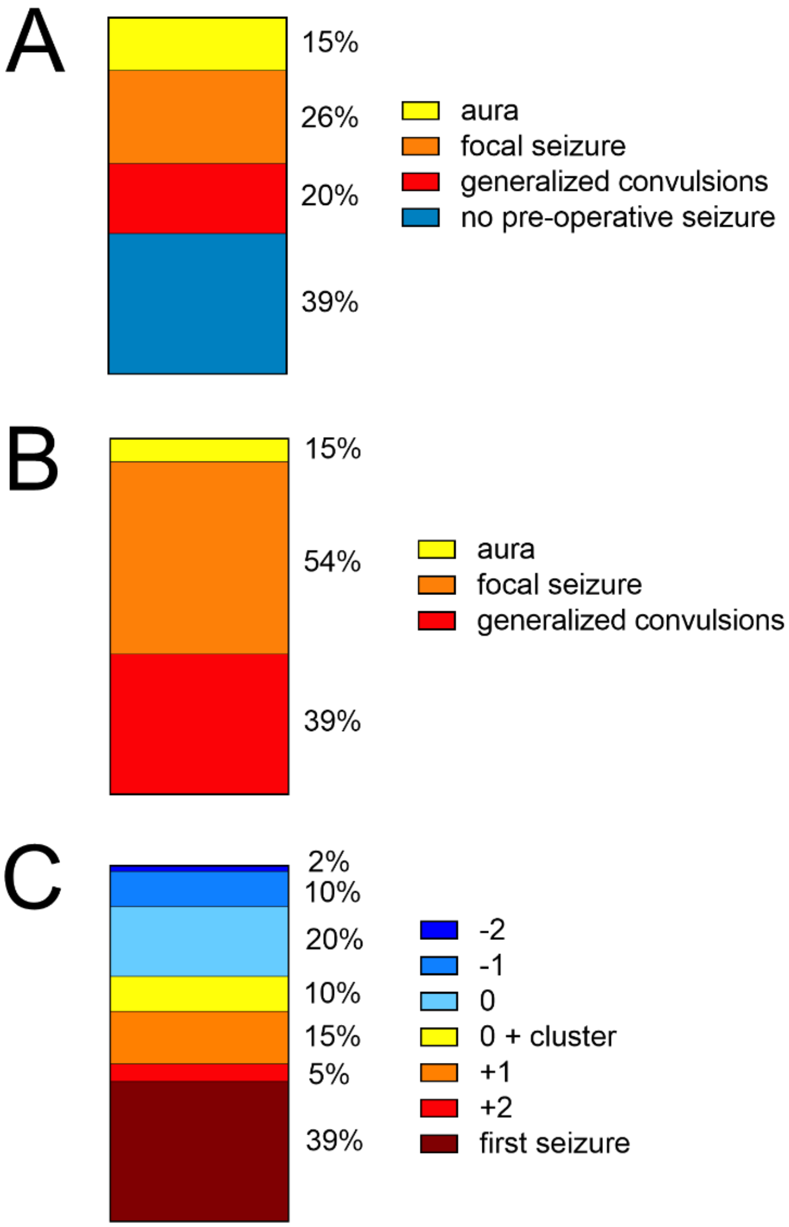

Supplemental Figure S2: Characteristics of postoperative seizures in patients with IDHwt GBM. (**A**) For patients with IDHwt GBM and post-operative seizures (n=61), preoperative seizures were categorized as aura (n=9), focal seizure (n=16), generalized convulsions (n=12), or no pre-operative seizures (n=24). (**B**) Postoperative seizures were categorized as aura (n=4), focal seizure (n=33) or generalized convulsions (n=24). (**C**) Changes in category for each patient's postoperative seizure compared to their preoperative seizure (e.g., "-2" means the preoperative seizure was a generalized convulsion, whereas the postoperative seizure was an aura.)

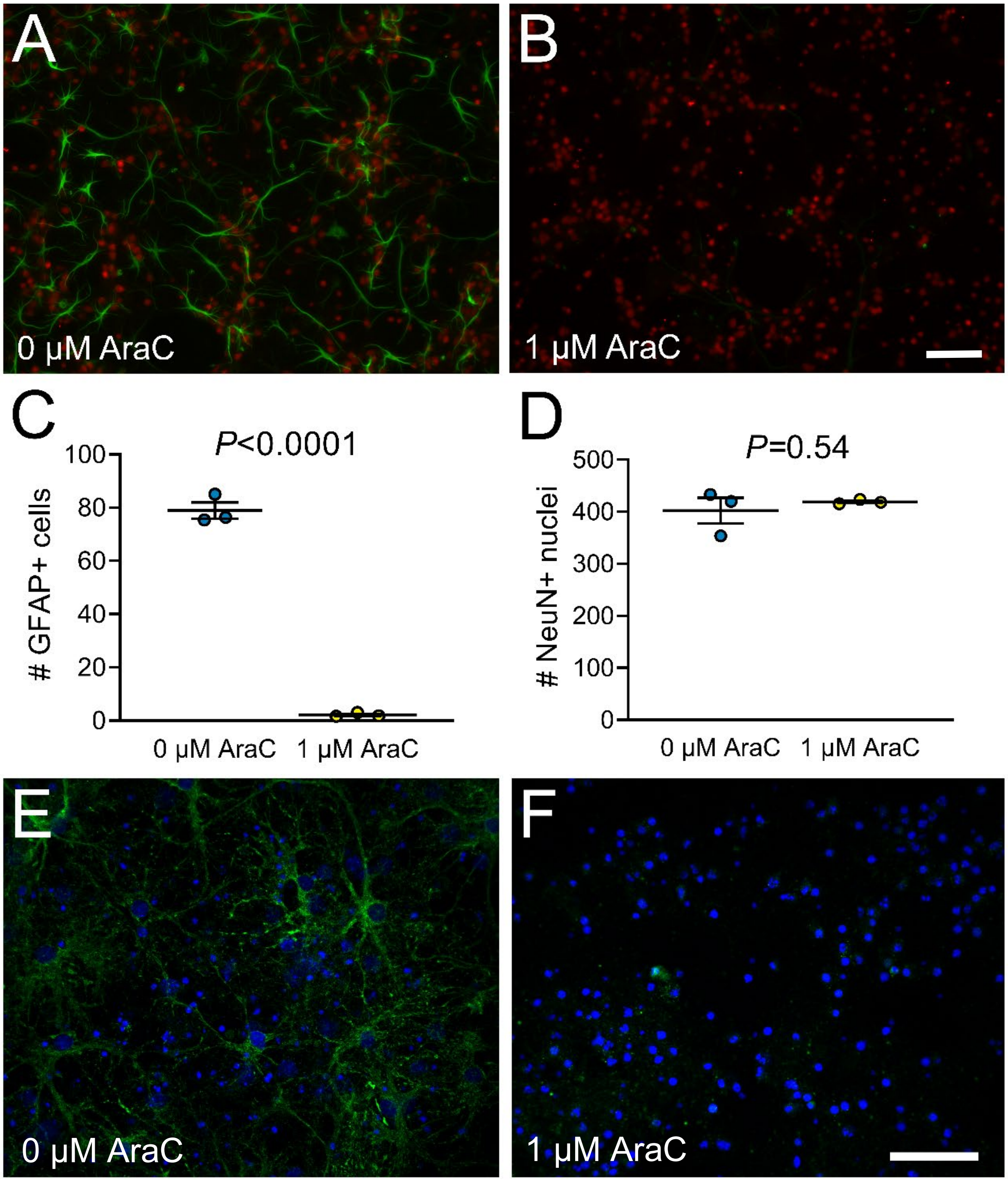

Supplemental Figure S3: **Generation of neuronal-glial cocultures versus neuron-only cultures**. Representative image of day 21 *in vitro* mouse neuron and glial co-culture treated with 0 μM AraC (**A**) or 1 μM AraC (**B**) on day 1 after plating, stained for NeuN (1:400, ab104224, red) and GFAP (1:1000, AB5804, green). Scale bar in (B) is 100 μm. Quantification of (**C**) number of GFAP-positive glial cells and (**D**) number of NeuN-positive nuclei. Bars represent mean ± SEM. Data were analyzed with unpaired t-test. Data points are the average quantification of five fields per replicate, n=3 replicates. (**E**) Representative image of day 21 *in vitro* mouse neuron and glial co-culture treated with 0 μM AraC (**A**) or 1 μM AraC (**B**) on day 1 after plating, stained for ALDH1L1 (1:500, ab177463). Scale bar in (F) is 100 μm.

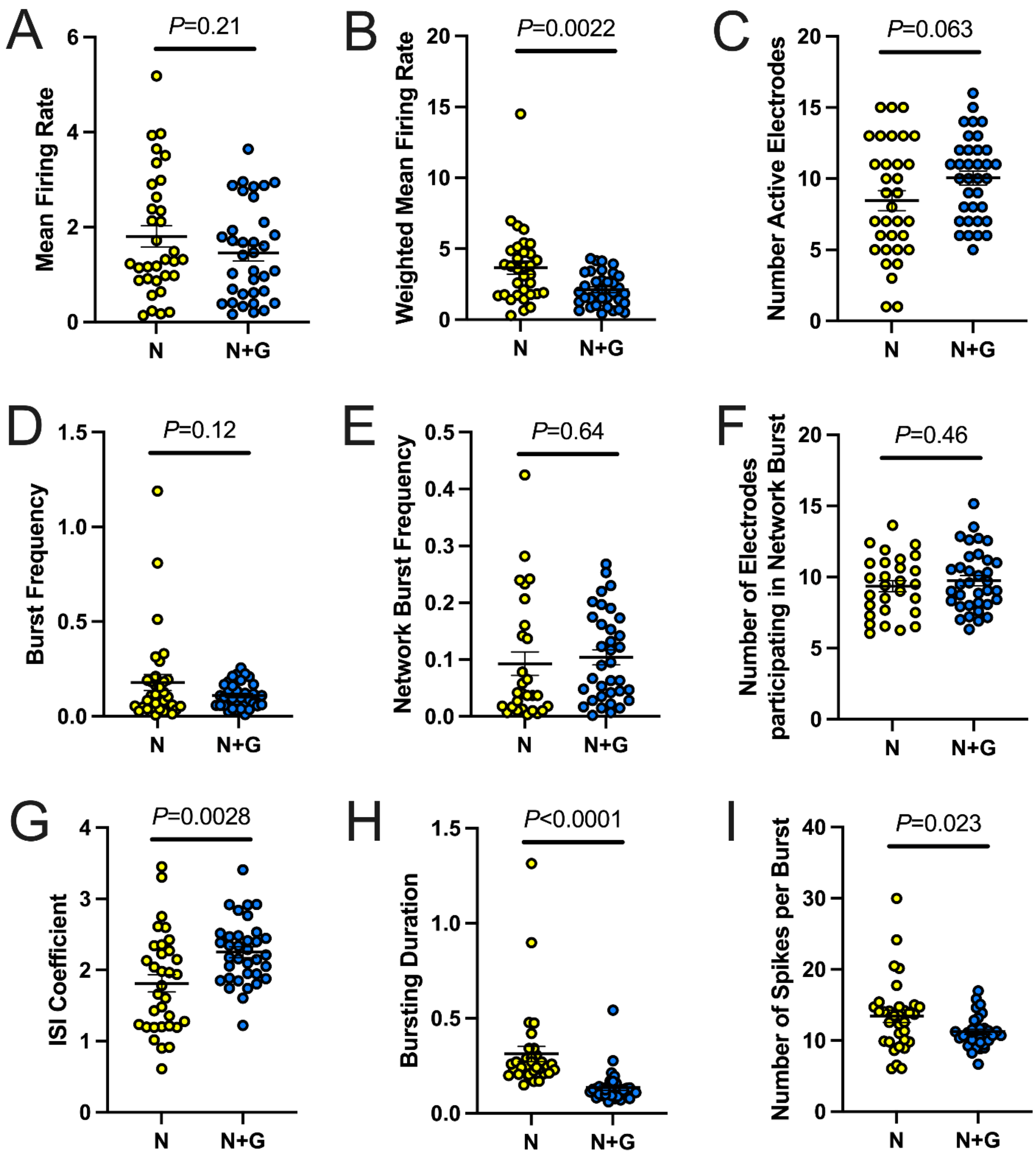

Supplemental Figure S4: Baseline firing patterns of neuronal-glial cocultures versus neuron-only cultures. Baseline firing patterns over 10 minutes of recording were measured for neuronal-glial cocultures (n=35) or neuron-only cultures (n=33). Bars represent mean ± SEM. **P*<0.05, ***P*<0.01, *****P*<0.0001, unpaired t-test. Abbreviations: N = neuron-only cultures, N+G = neuronal-glial cocultures.

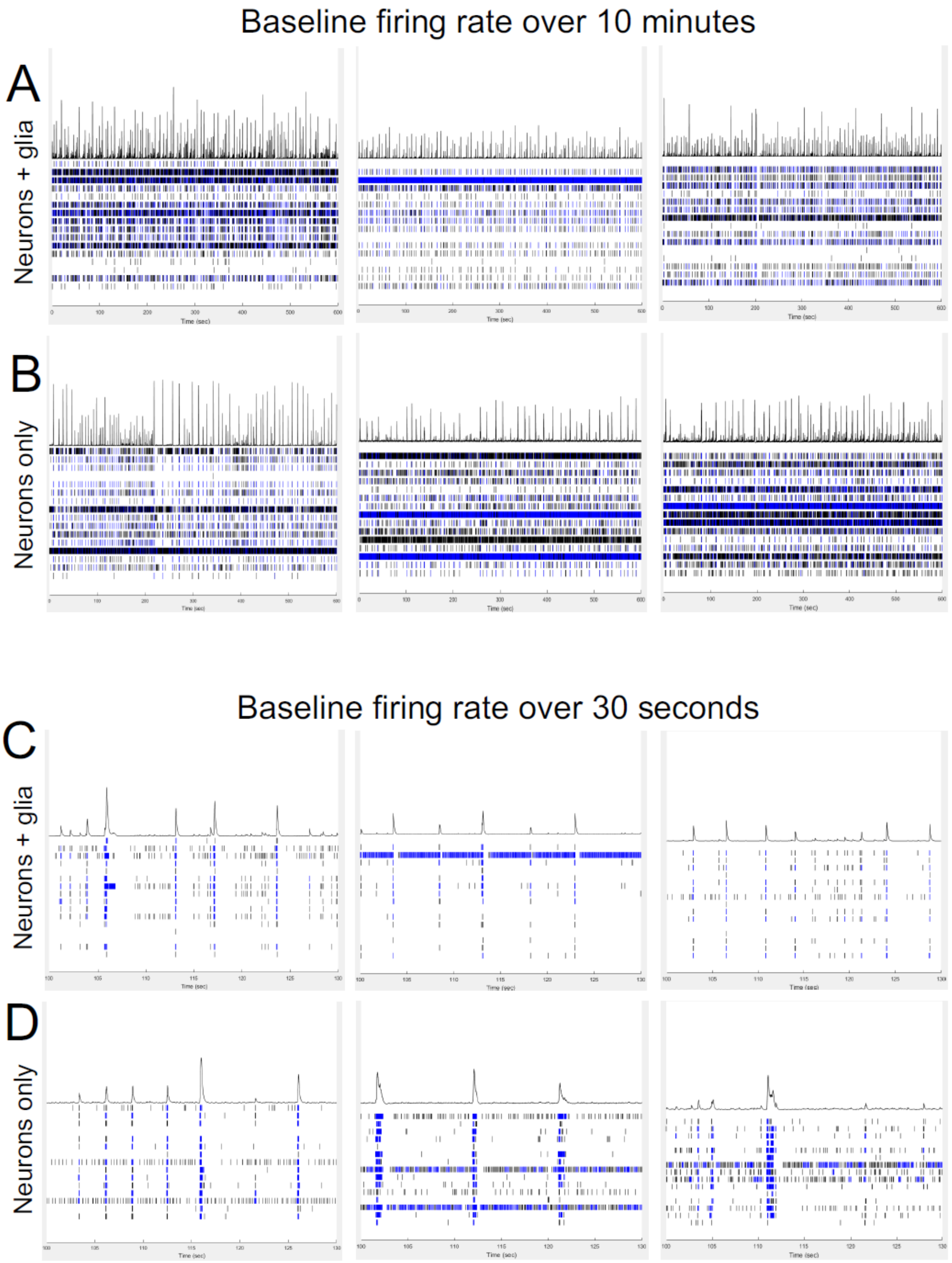

Supplemental Figure S5: Baseline raster plots of firing patterns of neuronal-glial cocultures versus neuron-only cultures grown on multi-electrode array plates. Three examples each of the entire 10-minute baseline recording period of (**A**) neuronal-glia cocultures and (**B**) neuron-only cultures, as well as three examples each of the same 30 second windows of (**C**) neuronal-glia cocultures and (**D**) neuron-only cultures. Each row encodes a given electrode within the 16-electrode array per well, and the histograms above the rows sums the firing activity across all electrodes at a given time.

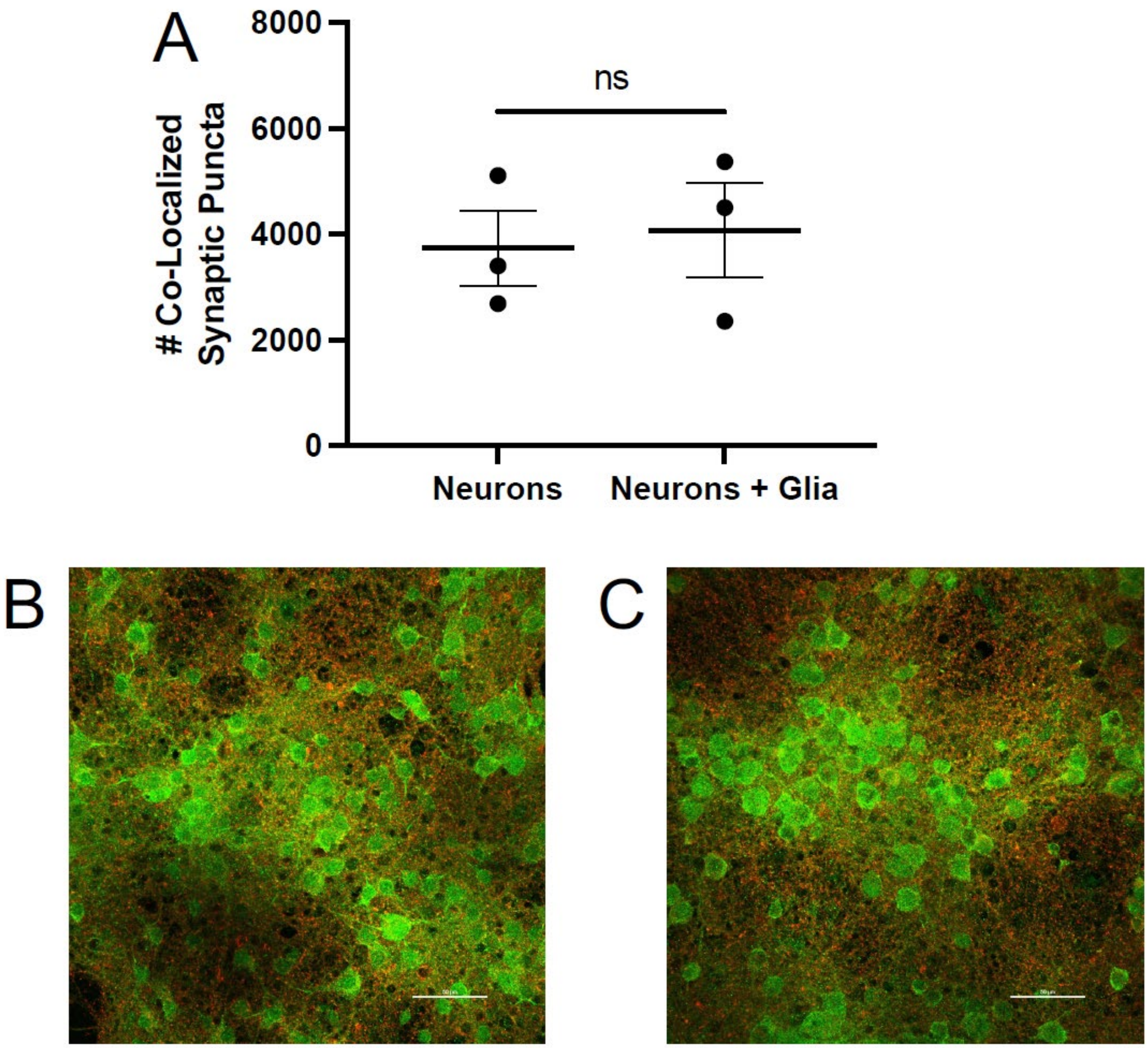

Supplemental Figure S6: Quantification of synaptic puncta in neuronal-glial cocultures versus neuron-only cultures. Neuronal-glial cocultures and neuron-only cultures were stained for PSD95 (1:200, ab18258, green) and Synaptophysin (1:200, MA1-213, red). (**A**) Quantification of co-localized PSD95 and synaptophysin. Bars represent mean + SEM. Data were analyzed with unpaired t-test. Data points are the average quantification of three fields per replicate, n=3 replicates. Representative images of (**B**) neuron-only and (**C**) neuronal-glial cocultures. Scale bar is 50 μm.

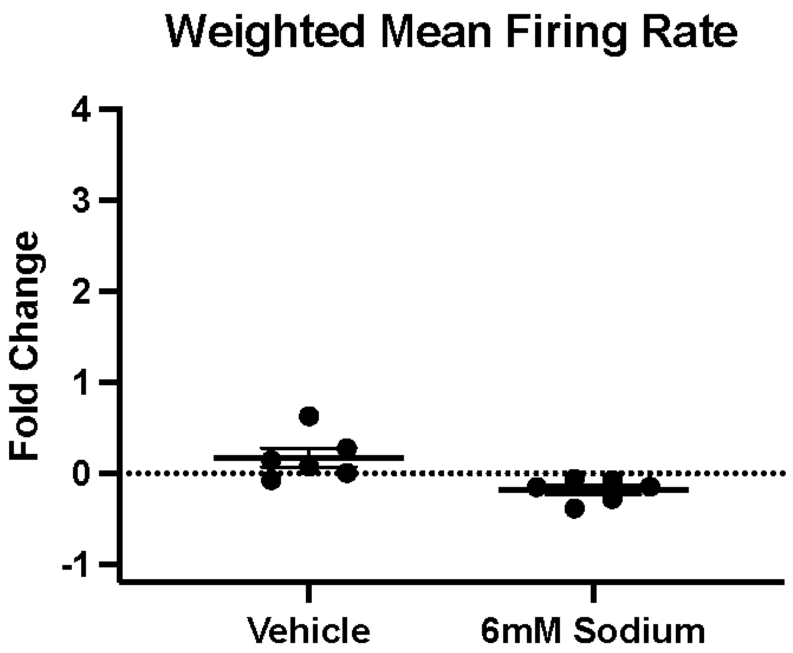

Supplemental Figure S7: Neuronal-glia cocultures with vehicle control or 6 mM Na^{+}. Neuronal-glia cocultures were treated with either vehicle control (n=6) or 6mM sodium (n=6). Bars represent mean ± SEM.

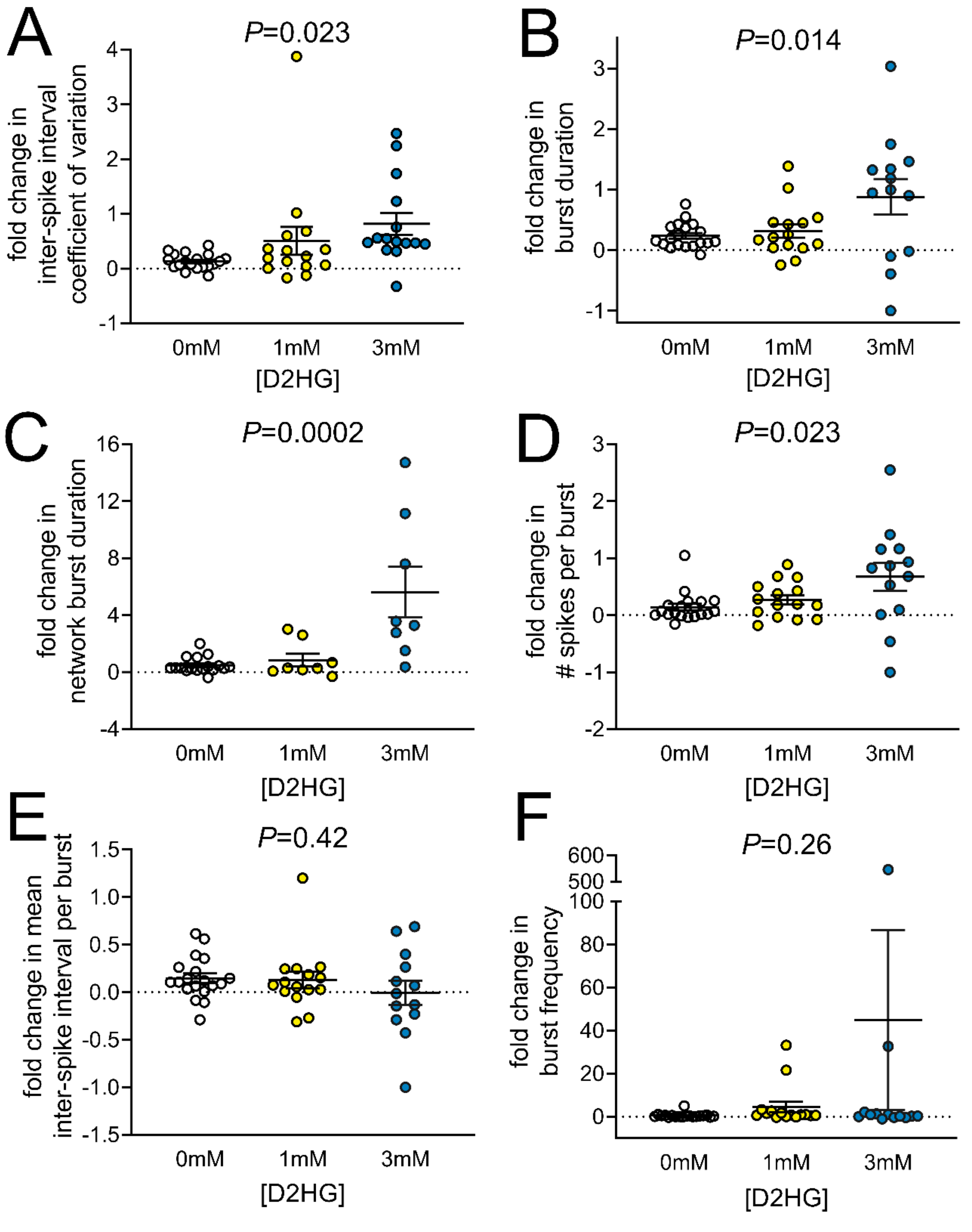

Supplemental Figure S8: **Effects of D2HG on firing characteristics of neuronal-glial cocultures**. **(A-F)** Day 21 mouse neurons cultured with glia had baseline firing characteristics recorded on a multi-electrode well plate for 5 minutes, and then treated with either vehicle (n=19), 1 (n=15), or 3 mM (n=13) D2HG. Fold change of firing characteristics for 5 minutes after treatment was calculated for each well. Bars represent mean ± SEM. Data were analyzed with unpaired t-test.

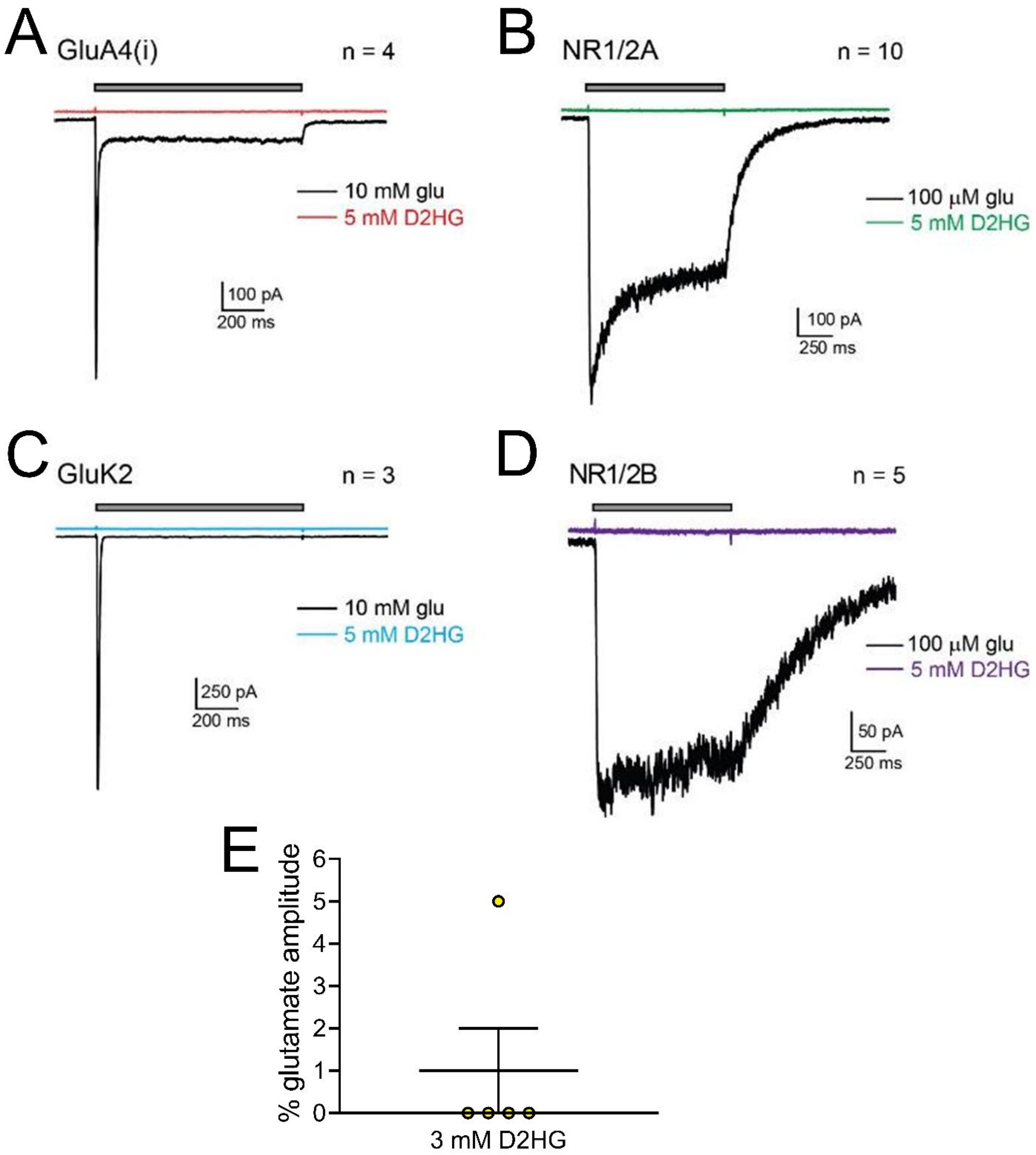

Supplemental Figure S9: **D2HG activity on ionotropic glutamate receptors and reuptake transporters.** (**A-D**) Whole-cell patch recordings were performed on multiple classes of ionotropic glutamate receptors that were transiently expressed in HEK293 cells. (**E**) Whole-cell patch recordings (n=5) were performed with excitatory amino acid isoform GLT1a expressed in HEK293 cells.

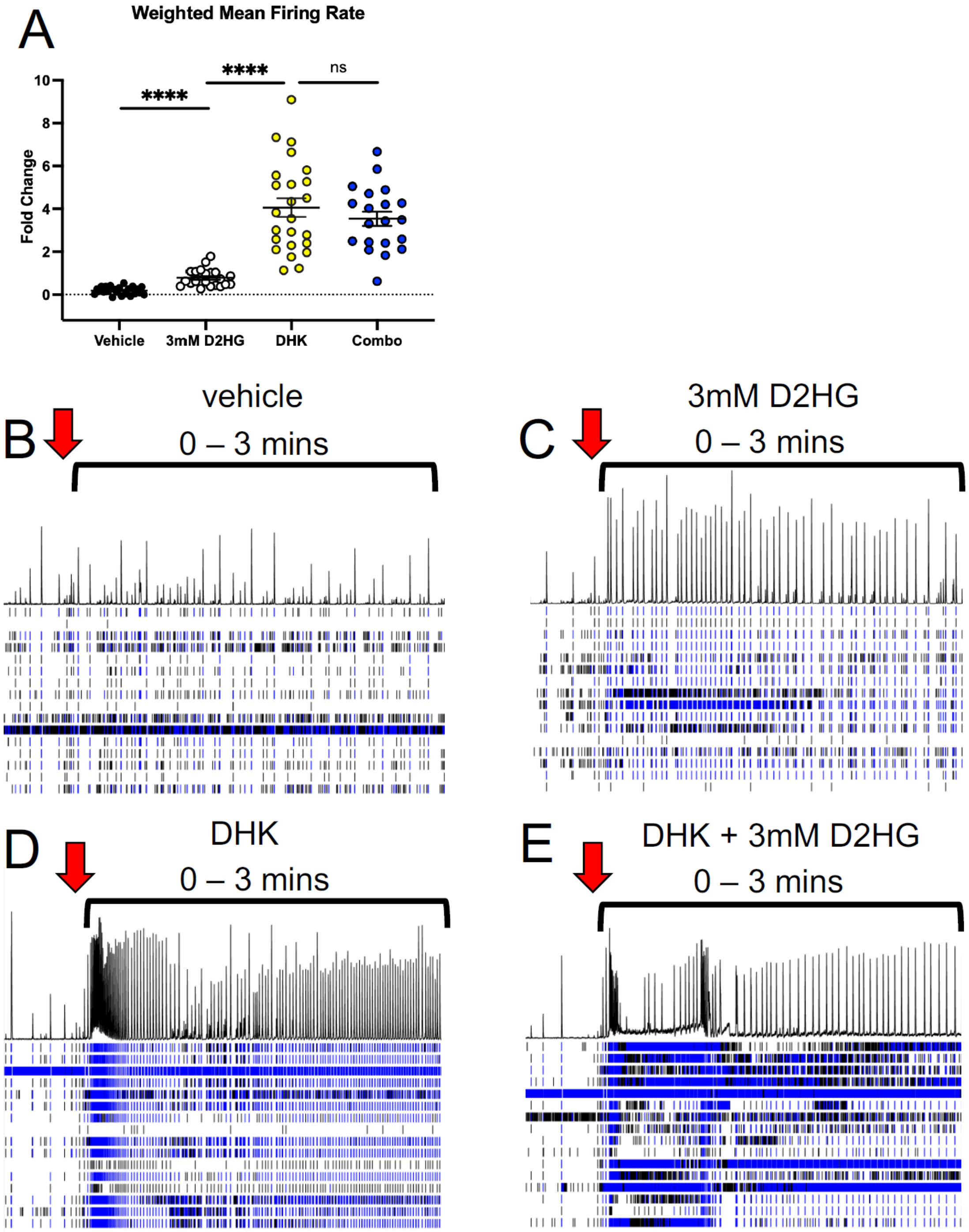

Supplemental Figure S10: **D2HG and dihydrokainate effects on neuronal firing. (A)** Day 21 mouse neurons cultured with glia had baseline weighted mean firing rate recorded on a multi-electrode well plate for 5 minutes, and then treated with either vehicle (n=23), 3 mM D2HG (n=21), 300 μM dihydrokainate (n=24), or 3mM D2HG and 300 μM dihydrokainate (n=20). Fold change of weighed mean firing rate for 5 minutes after treatment was calculated for each well. Bars represent mean ± SEM. ****P<0.0001, unpaired t-test. (**B-E**) Representative raster plot of neuron and glia co-cultures treated with (**B**) vehicle, (**C**) 3mM D2HG, (**D**) 300 μM dihydrokainate, or (**E**) 3mM D2HG and 300 μM dihydrokainate. Treatment was initiated at the timepoint indicated by the red arrows.

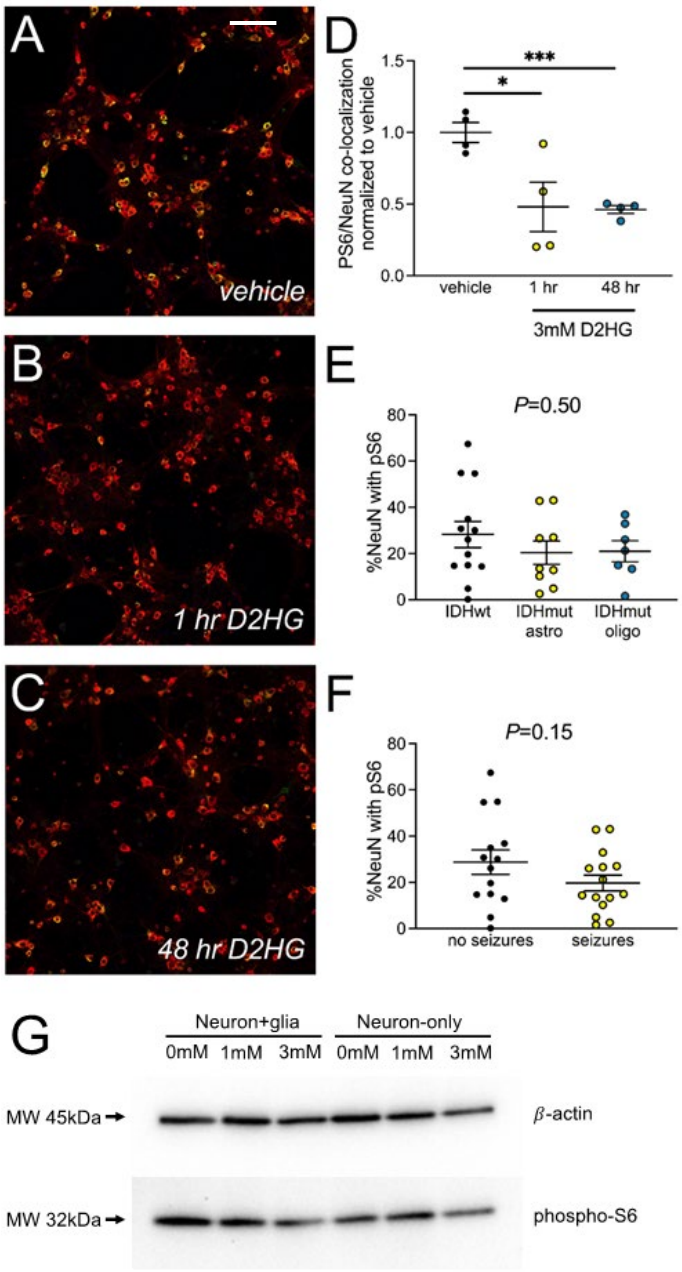

Supplemental Figure S11: mTOR pathway activity in response to D2HG and in IDHmut gliomas. (**A-D**) Mouse neurons and glia were co-cultured on coverslips and treated either with (**A**) vehicle or (**B**) 3mM D2HG on day 21 an hour before fixing, or with (**C**) 3mM D2HG on day 19 48 hours before fixing. Fixed co-cultures were stained for NeuN (1:400, ab104224, red) and phosphoS6 (1:1000, 2211, green). Scale bar in (A) is 100 μm. (**D**) Quantification of co-localized NeuN and phosphoS6. Bars represent mean ± SEM. Data points are the average quantification of five fields per replicate, n=4 replicates. *P<0.05, ***P<0.001, unpaired t-test. (**E** and **F**) Patient-derived tissues from newly diagnosed adult-type diffuse gliomas were stained for NeuN and phosphoS6. Staining and imaging were performed independently at two separate institutions, and the results were pooled. Bars represent mean ± SEM. Data were analyzed with unpaired t-test. (**G**) Neurons and glia co-cultures or neurons alone were treated with either vehicle (0mM), 1mM D2HG, or 3mM D2HG for 5 minutes, then cells were collected to analyze protein expression by western blot.

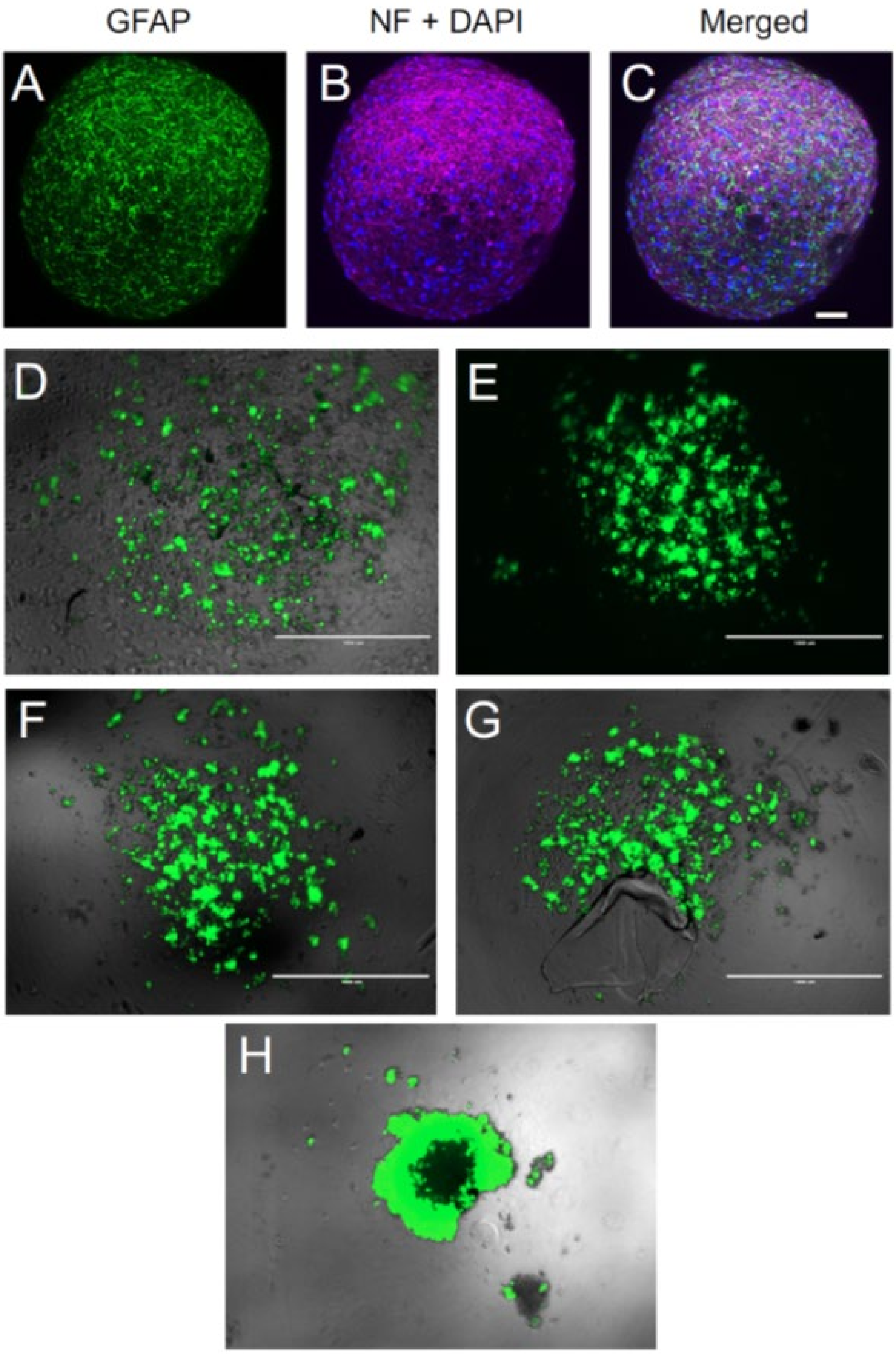

Supplemental Figure S12: Glioma infiltrate into spheroids. (**A-C**) Mature cortical spheroids are comprised of GFAP-positive astrocytes (GFAP: 1:1000, Z033429-2, green) and Neurofilament-positive neurons (NF: 1:1000, SMI-312, magenta). Scale bar = 50 μm. (**D-F**) After 24 hours of culture in U-bottom wells, GFP-labeled glioma loosely aggregate in the bottom of the well (3 representative images), (**G**) and do not infiltrate nor aggregate around a small ball of agarose. They do closely aggregate and infiltrate into spheroids (**H**). Scale bars = 1000 μm.

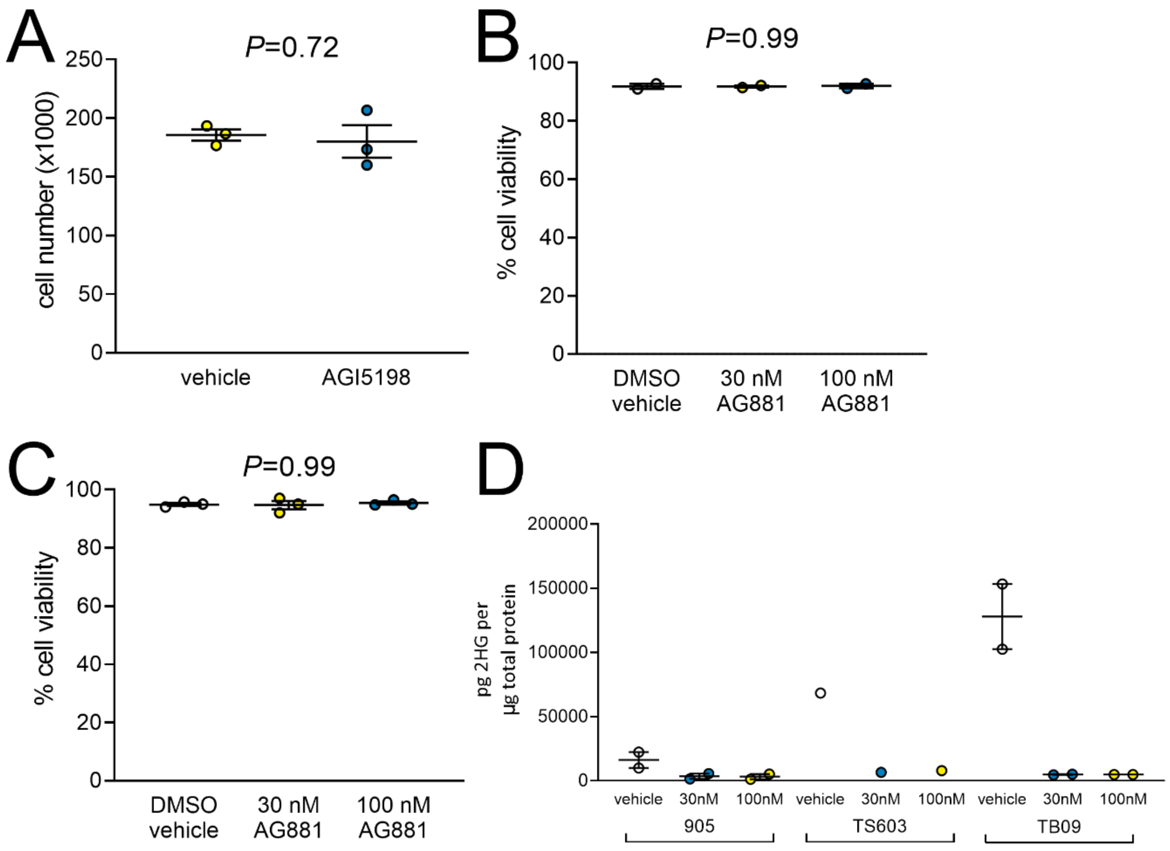

Supplemental Figure S13: Effect of IDHmut inhibitors on IDHmut glioma cell growth and 2HG production. (**A**) Patient-derived IDHmut glioma TB09 cells (n=6 technical replicates, n=3 biological replicates) were treated with either vehicle or 1.5 μM AGI5198 for two days, and then cell count was performed. Bars represent mean ± SEM. Data were analyzed with unpaired t-test. (**B**) Patient-derived IDHmut glioma 905 (n=4 technical replicates, n=2 biological replicates) and (**C**) IDHmut mouse glioma (n=4 technical replicates, n=3 biological replicates) were treated with either vehicle, or 30 nM or 100 nM AG881 for two days, and then cell viability was assessed. Bars represent mean ± SEM. Data were analyzed with one-way ANOVA. (**D**) Patient-derived IDHmut glioma cells were treated with either vehicle, or 30 nM or 100 nM AG881 for two days, and then 2HG levels were assessed via liquid chromatography mass spectrometry (2HG enantiomers could not be distinguished via liquid chromatography mass spectrometry).

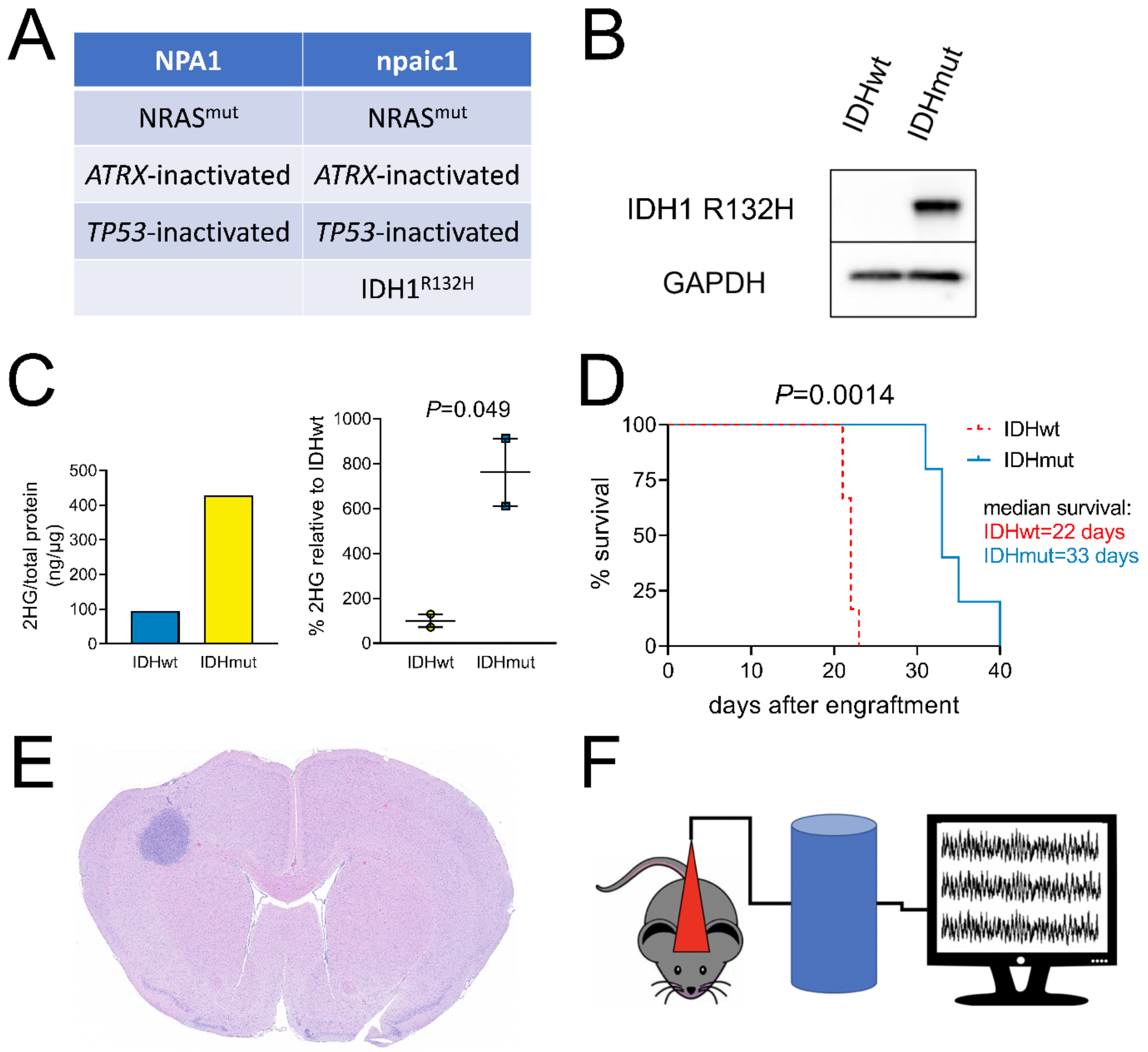

Supplemental Figure S14: Characteristics of IDHwt and IDHmut glioma seizure models. (**A**) Molecular alterations in the isogenic Sleeping-Beauty-engineered mouse glioma lines. NPA1 = IDHwt, npaic1 = IDHmut. (**B**) Western blot probing for R132H mutant IDH1 protein (DIA-H09, 1:500). **(C)** IDHwt and IDHmut glioma was cultured for two days, and then cell pellets were collected for assessment of intracellular 2HG levels via mass spectrometry, or conditioned media was collected for assessment of extracellular 2HG levels via mass spectrometry (n=2 technical replicates, n=2 biological replicates). Intracellular 2HG levels were normalized to total protein of each cell pellet, and extracellular 2HG levels were normalized relative to the average 2HG level in the IDHwt samples. Bars represent mean ± SEM. Data were analyzed with unpaired t-test (**D**) C57BL/6 were engrafted with IDHwt (n=6) or IDHmut (n=5) mouse glioma and monitored for survival. Data were analyzed with log-rank (Mantel-Cox) test. (**E**) Representative H&E image of engrafted IDHmut glioma. (**F**) Schematic of EEG monitoring set-up.

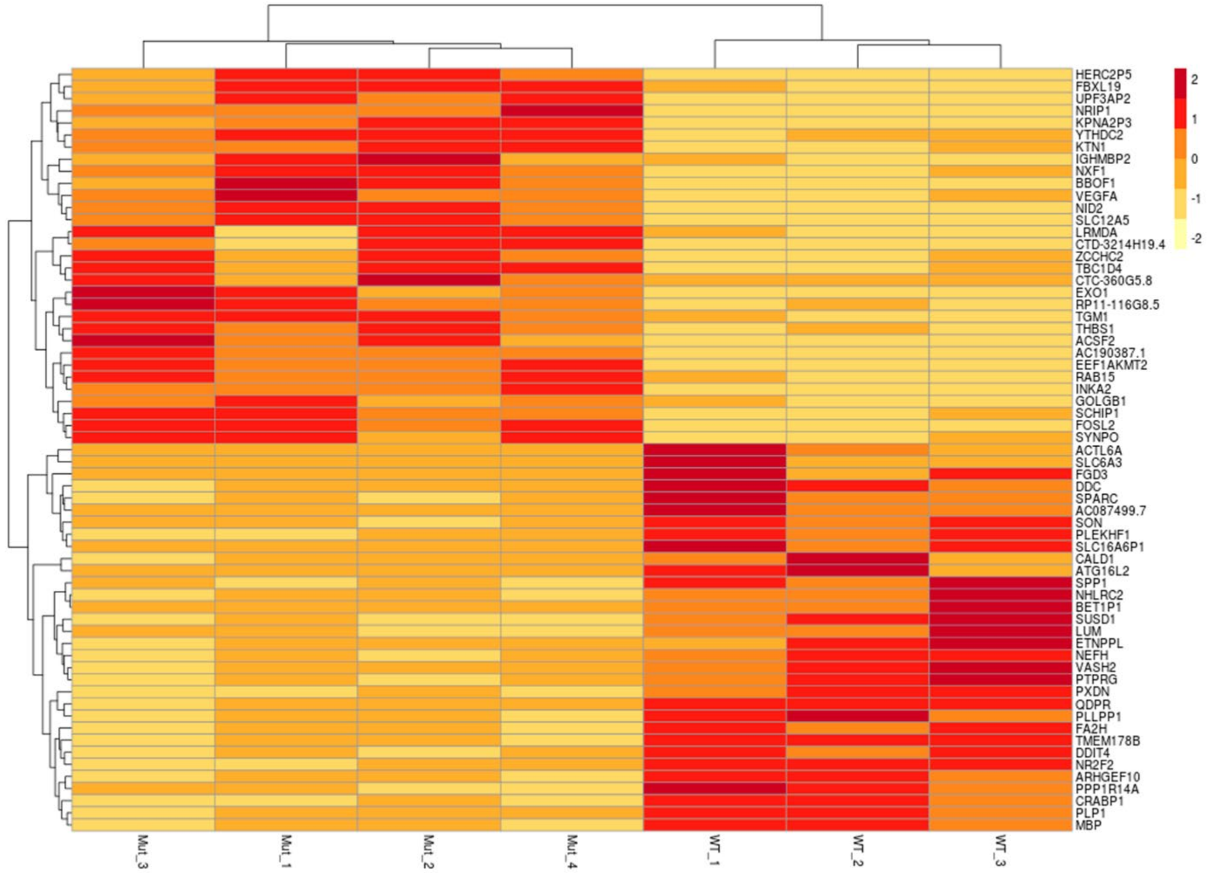

Supplemental Figure S15: RNA-Seq profiles of peritumoral tissues around IDHwt vs IDHmut glioma. C57BL/6 mice were engrafted with either IDHwt (n=3) or IDHmut (n=4) glioma and euthanized on day 12. Tumor was removed, and peri-tumoral tissue was collected and prepared for RNA-Sequencing. Differentially expressed genes between IDHwt and IDHmut are displayed, with log2 fold change displayed for each sample.
